# Integrating Transcriptomics and Free Fatty Acid Profiling Analysis Reveal Cu Induces Shortened Lifespan and Increased Fat Accumulation and Oxidative Damage in *C. elegans*

**DOI:** 10.1155/2022/5297342

**Published:** 2022-08-16

**Authors:** Ying Zhang, Qian Zhou, Lu Lu, Chao Zhao, Hu Zhang, Ran Liu, Yuepu Pu, Lihong Yin

**Affiliations:** Key Laboratory of Environmental Medicine Engineering, Ministry of Education of China; School of Public Health, Southeast University, Nanjing 210009, Jiangsu, China

## Abstract

Nowadays, human beings are exposed to Cu in varieties of environmental mediums, resulting in health risks needing urgent attention. Our research found that Cu shortened lifespan and induced aging-related phenotypes of *Caenorhabditis elegans* (*C. elegans*). Transcriptomics data showed differential expression genes induced by Cu were mainly involved in regulation of metabolism and longevity, especially in fatty acid metabolism. Quantitative detection of free fatty acid by GC/MS further found that Cu upregulated free fatty acids of *C. elegans*. A mechanism study confirmed that Cu promoted the fat accumulation in nematodes, which was owing to disorder of fatty acid desaturase and CoA synthetase, endoplasmic reticulum unfolded protein response (UPR^ER^), mitochondrial membrane potential, and unfolded protein response (UPR^mt^). In addition, Cu activated oxidative stress and prevented DAF-16 translocating into nuclear with a concomitant reduction in the expression of environmental stress-related genes. Taken together, the research suggested that Cu promoted aging and induced fat deposition and oxidative damage.

## 1. Introduction

In recent decades, environmental pollution has become a globalization problem [1, 2]. Environment pollution would induce water quality problems, especially excessive heavy metals [3, 4]. Copper (Cu) has attracted widespread attention due to its utilization for water pipes since 19th century [5]. As an essential trace element, Cu is needed in trace amounts in human and animal [6]. The daily intake of Cu ranges from 0.7 to 3.0 mg/day [7]. Several foods are rich in Cu, including meats, seafood, kidney and liver, mushrooms, and nuts, which is a sufficient supply for daily intake. Thus, the Cu content in the environment and the Cu level in drinking water are particularly important.

Due to favorable mechanical properties, Cu was used extensively in drinking water distribution systems [8, 9]. However, metals could not escape the effects of corrosion. The release of Cu particles or ions into the drinking water would cause human health risk [10]. Except for Cu pipes, Cu mining and use of Cu-containing fungicides might affect soil Cu concentration, then directly or indirectly alter the Cu content in animals and plants, finally contribute to excessive Cu intake of human [11, 12]. As the Paracelsus principle pointed, “the dose makes the poison” [13].

Cu participates in the synthesis of various proteins and enzymes and is very important in tissue structure and various enzymatic reactions. For example, Cu is involved in the formation of connective tissue, myelin, and formation of melanin pigment in the skin, hair, and eyes [14–16]. Cu also plays as an enzymatic cofactor, such as bounding to ceruloplasmin for oxidase activity and system circulation [17], and constitutes cytochrome c oxidase to participate in the respiratory chain in mitochondria [18]. In addition, Cu is a component of cytochrome P450 monooxygenase, dopamine-beta-hydroxylase, and copper, zinc-superoxide dismutase (Cu, Zn-SOD) [18–20]. Cu is found in cells and tissues throughout the body, especially abundant in the liver and brain [7]. Therefore, Cu overload induced-malfunctions contributes to the development of hepatic, neurological, and other disorders.

The US Environmental Protection Agency (EPA) had proposed the Optimized corrosion control treatment (OCCT) to carry out the lead and copper rule (LCR) [21]. However, some water systems still exceeded the action level of Cu [22]. Not to mention the hazards of exceeding the upper limit, even the upper limit of the drinking water regulations might not be safety. Thus, this study was focus on two standards for drinking water quality L set up by WHO and 1 mg/L set up by China (GB 5749—2006). The long-term chronic effect of Cu on human health has not been well studied. Actually, chronic effect could also occur when exposed to Cu during a period of time.

*Caenorhabditis elegans* (*C. elegans*) is an outstanding experimental system, due to its small size, rapid life cycle, transparency, and well-annotated genome [23]. *C. elegans* could provide data with aging, reproductive, endocrine, sensory, and neuromuscular systems [24]. In addition, *C. elegans* has the high conservation of genes and signaling pathways with mammals [25]. Thus, this study is aimed at exploring the effects of Cu exposure on the health, lifespan, and the underlying mechanism of model organisms *C. elegans*.

## 2. Experimental Procedures

### 2.1. *C. elegans* Strains and Culture Methods

The wild-type N_2_ strain, CF1553 muIs84[(pAD76) *sod-3*p::GFP+rol-6(su1006)], CF1038 *daf-16* (mu86), TJ356 zIs356[*daf-16*p::daf-16a/b::GFP+rol-6(su1006)], SJ4005 zcIs4[HSP-4::GFP], and SJ4100 zcIs13[HSP-6::GFP] were purchased from the *Caenorhabditis* Genetics Center (CGC, Minneapolis, MN 55455, USA). *C. elegans* was grown using *E. coli* strain OP50 as a food source on the nematode growth medium (NGM) at temperature of 20°C. NGM preparation with 90 mm Petri dishes (Corning, USA) and *E. coli* OP50 preparation were performed following the recommendations of the CGC (Minneapolis, MN, USA) [26].

### 2.2. Cu Preparation and Treatment

CuSO_4_·5H_2_O (purity > 98%, CAS No. 7758-99-8, Sigma-Aldrich, USA) was suitable for *C. elegans* culture. The CuSO_4_·5H_2_O was dissolved in K-medium and formulated into a 1 g/L stock solution. As reported, guidelines for drinking water quality issued by the World Health Organization (WHO, fourth edition) and China (GB 5749-2006, China) stipulate the guideline value of Cu were 2.0 mg/L and 1.0 mg/L [27, 28]. Thus, Cu concentrations were designed as 0 (K-medium control), 1, and 2 mg/L.

Synchronized L1 stage larvae were cultivated for 24-28 h to obtain L4 stage. L4 stage larvae were treated with Cu (control, 1 and 2 mg/L) for 24 h in 35 mm NGM plate (Corning, New York, USA), 200 *μ*L treatment solution/plate. Subsequently, the following experiments were conducted.

### 2.3. Measurement of Lifespan, Morphological, and Behavioral of *C. elegans*

#### 2.3.1. Lifespan Assay

Lifespan was determined by counting worms daily until all died [29]. The worms were confirmed dead if no movements were observed by touching with a platinum wire at the tail and the head. In brief, synchronized N_2_ worms and CF1038 worm were raised to the L4 stage and then were treated with Cu and control on 35 mm NGM plates (with 30 nematodes/plate, total 360 worms/condition, three independent experiments were carried out). After Cu treatment until adult, the *C. elegans* were transferred to new clean plate and for recognizing the viability of worms under a stereomicroscope every day. Floxuridine (FUdR) was not used to avoid progeny production, and the animals were transferred every day during the fertile period.

#### 2.3.2. Body Growth Index Assay

Body length and width were obtained to evaluate the development. Body growth indexes were detected using Zeiss microscope (Zeiss AX10, Germany) to capture the worms at least 20 for each group.

#### 2.3.3. Pharyngeal Pumping and Defecation Assay

Pharyngeal pumping and defecation interval are age-related changes [29]. Pumping frequency is determined by counting olfactory bulb pumping over a 30 s timespan [30]. Defecation interval is determined as the period between the two posterior intestinal muscles [30]. At least 20 worms were counted for each group.

All experiments were independently repeated three times.

### 2.4. Sample Preparation, Library Construction, and RNA Sequencing

L4 stage larvae were treated with Cu (control, 1 and 2 mg/L) until adult. A total of 3 treatment groups were obtained (L4-0, L4-1, and L4-2) each with three biological replicates. *C. elegans* were collected to extract total RNA using TRIzol reagent kit (Invitrogen, Carlsbad, CA, USA) according to the protocol. Then, sample preparation, library construction, and RNA sequencing were conducted according to previous description [31]. RNA sequencing was performed using Illumina HiSeq^TM^ 6000 by Gene Denovo Biotechnology Co., Ltd. (Guangzhou, China). The experimental details were descripted in the supplementary information (SI).

The raw RNA-seq data of the study have been deposited in NCBI SRA (http://www.ncbi.nlm. http://nil.gov/sra) with the accession number SRP323540.

### 2.5. Bioinformatics Analysis

DESeq2 software was performed to analyze differential expression genes (DEGs) [32]. Gene Ontology database (http://www.geneontology.org/) and Kyoto Encyclopedia of Genes and Genomes (KEGG) database were used for GO term analyses and pathway analyses. The experimental details were described in the SI.

### 2.6. Fat Staining

#### 2.6.1. Oil Red O Staining

Oil Red O staining solution (Solarbio, Beijing, China) was used for lipid detection. Cu-treated worms were washed with M9 solution and fixed in 4% paraformaldehyde/M9 for 30 min. Oil Red O staining solution was performed for lipid detection according protocol. Cu-treated worms were washed with M9 solution and fixed in 4% paraformaldehyde/M9 for 20 min. Then, discard the fixative and wash with M9 three times, dehydrate with 60% isopropanol for 5 min, and stain with Oil Red O stain for 15 min. Wash 3 times with M9, add Oil Red O buffer for 1 minute, and mounted to 2% agarose padded slides. Zeiss microscope (Zeiss AX10, Germany) was used for worms photographing. ImageJ was for quantifying intensity of Oil Red O staining.

#### 2.6.2. Nile Red Staining

Nile Red (Sigma-Aldrich, USA) was formulated as a 0.5 mg/L storage solution and stored without light. Cu treated, washed, fixed, and dehydrated were the same as Oil Red O staining. Nile Red storage solution was diluted (1 : 250) and for nematodes staining the in the dark for 30 minutes. Then, worms were washed 3 times, anesthetized with 50 mM levamisole, mounted to 2% agarose padded slides, and observed lipid droplets under a fluorescence microscope (Zeiss AX10, Germany). Red fluorescent was quantified by ImageJ.

All experiments were independently repeated three times, at least 30 nematodes/group were examined.

### 2.7. Detection of ROS, MMP (*Δψ*m), UPR^mt^, UPR^ER^, and DAF-16 Nuclear Accumulation

#### 2.7.1. ROS and MMP (*Δψ*m)

Fluorescent probe H2DCFDA (KeyGEN, Nanjing, China) was used to detected reactive oxygen species (ROS) level, and JC-1 probe (Invitrogen, USA) was used to tested mitochondrial membrane potential (MMP, *Δψ*m) according to the manufacturer's instructions. The experimental details were described in the supplementary text.

#### 2.7.2. The Image Analysis of SOD-3::GFP, HSP-6::GFP, and HSP-4::GFP

SOD-3::GFP, HSP-6::GFP, and HSP-4::GFP were response to ROS production, mitochondrial unfolded protein response (UPR^mt^) signal, and endoplasmic reticulum stress (ER stress), respectively. Thus, CF1553 (SOD-3: GFP), SJ4100 (HSP-6::GFP), and SJ4005 (HSP-4::GFP) were performed to detected of antioxidation defense, UPR^mt^, and endoplasmic reticulum unfolded protein response (UPR^ER^) [33]. The Cu treatment of used strains were the same as the N_2_ strain; then, semiquantification of fluorescent signals was determined by ImageJ.

#### 2.7.3. Analysis of the Subcellular Localization of the DAF-16

As described, TJ356 (the strain expressing DAF-16 fused to GFP) was used to observe the localization the transcription factor DAF-16 [34]. L4 stage TJ356 worms were firstly transferred to NGM plates without OP50 for starving 16 h to induce nuclear DAF-16 localization. Then, this worms were exposed with 0, 1, and 2 mg/L Cu in NGM plates for 24 h at 20°C. The subcellular DAF-16 localization was analyzed by fluorescence microscopy (Zeiss). The localization of the DAF-16::GFP fusion protein of TJ356 worms were classified into nuclear, cytosolic, and both nuclear and cytosolic three categories.

Experiments 2.7.1 to 2.7.3 were independently repeated three times, at least 30 nematodes/group were examined.

### 2.8. Real-Time Quantitative Polymerase Chain Reaction (RT-qPCR)

Gene expression was conducted by RT-qPCR using Gradient PCR instrument (Eppendorf, Germany), StepOne Plus (ABI, USA), and SYBR Green Master Mix (Takara, Tokyo, Japan). Briefly, RNA of *C. elegans* was reversed transcription to cDNA; then, quantitative PCR was performed according to the protocols. Primer sequences were listed in Table S1. Expression levels of target genes were evaluated by the 2^-*ΔΔ*Ct^ method. Experiments were independently repeated three times.

### 2.9. Metabolite Extraction and GC-MS Free Fatty Acid Analysis

L4 stage larvae were treated with Cu (control, 1 and 2 mg/L) until adult. A total of 3 treatment groups were obtained (L4-0, L4-1, and L4-2) each with five biological replicates. The N_2_ worms were washed for 5 times and taken into the 1.5 mL EP tubes; then, worms were conducted according to previously described methods [35]. The GC-MS analysis was performed using an Agilent 7890B gas chromatograph system (Agilent, California, USA) coupled with an Agilent 5977B mass spectrometer (Agilent, California, USA). In this study, 31 peaks were detected and 31 metabolites were left after relative standard deviation denoising. Then, the missing values were filled up by the median value. The final dataset containing the information of peak number, sample name, and normalized peak area was imported to SIMCA16.0.2 software package (Sartorius Stedim Data Analytics AB, Umea, Sweden) for multivariate analysis. Data was scaled and logarithmic transformed to minimize the impact of both noise and high variance of the variables. After these transformations, PCA (principle component analysis), an unsupervised analysis that reduces the dimension of the data, was carried out to visualize the distribution and the grouping of the samples. 95% confidence interval in the PCA score plot was used as the threshold to identify potential outliers in the dataset. The experimental details could be found in the SI.

### 2.10. Data Analysis

SPSS 23 (SCR: 002865) and the GraphPad Prism 9 (SCR: 002798) software were used to conduct data analysis. The statistics were described as mean ± SD from three independent experiments. The univariate analysis of variance (ANOVA) was performed to analyze the differences among multigroup, followed by Dunnett's test to identify significant difference between treatment and control. Kaplan-Meier survival analysis with log-rank test were used to analyze survival of the worms. ImageJ (SCR: 003070) was used for quantitative of fluorescence intensity.

## 3. Results

### 3.1. The Roles of Cu Exposure in Aging, Growth, and Behavior in *C. elegans*

**Figure 1:**
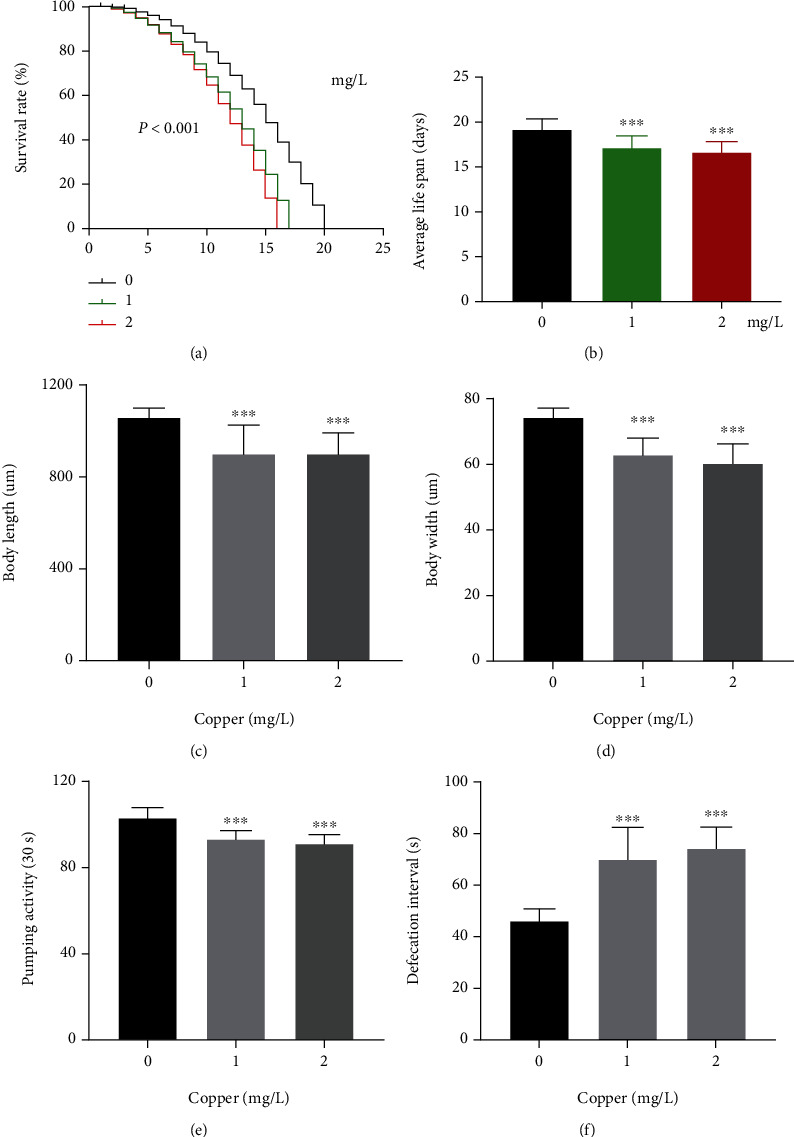
The roles of Cu exposure in aging, growth, and behavior in *C. elegans*. (a) Survival of *C. elegans* with 0, 1, and 2 mg/L Cu, 30 nematodes/plate, total 360 worms/condition. (b) Average lifespan was shortened, *n* ≥ 20/condition. (c) Body length and (d) body width was inhibited, *n* ≥ 20/condition. (e) Pharyngeal pumping frequency was decreased, *n* ≥ 20/condition. (f) Defecation intervals were prolonged (^∗∗∗^*P* < 0.001). Bars represent means ± SD, *n* = 3 independent experiments.

The L4 stage worms were treated with Cu to adult then were observed the survival until all worms died. The results showed lifespan and average lifespan of N_2_ worms were gradually decreased with Cu exposure (Figures 1(a) and 1(b)). Cu could induce negative effect on growth of *C. elegans*, manifested by shortening of body length and width (Figures 1(c) and 1(d)). In addition, body movement was detected. The results showed that Cu inhibited the pumping rate and extended defecation interval in a dose-dependent manner (Figures 1(e) and 1(f)), implying the promoting aging effect of Cu in *C. elegans.*

### 3.2. Identification of Differentially Expressed Genes (DEGs)

**Figure 2:**
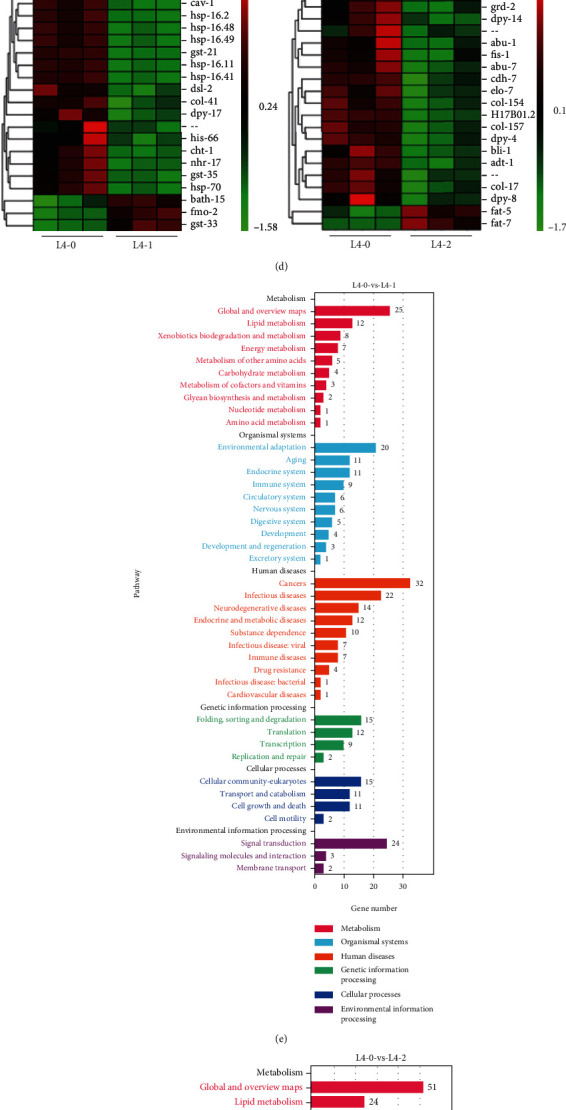
Identification of differentially expressed genes (DEGs) and pathway analysis. (a) Pearson correlation between samples. (b) Hierarchical clustering diagram of DEGs in the 0-VS-1 group and 0-VS-2 group. (c) Number of DEGs in 0-VS-1 group and 0-VS-2 group. (d) Heat-map of top 20 DEGs in the 0-VS-1 group and 0-VS-2 group. (e, f) KEGG pathway enrichment of Cu-exposed *C*. *elegans.*

RNA-sequencing was performed on three groups with 3 biological replicates each group of *C. elegans* and finally to construct a total of 9 cDNA libraries. Reads were obtained from the sequencing machines and were further filtered by fastp (version 0.18.0) following protocol [36]. Sample expression violin plot showed maximum, minimum, and median of the 9 sample genes were basically the same (Figure S1). Correlation of two parallel experiments provided the evaluation of the reliability of experimental results as well as operational stability. Correlation analysis showed that the correlation of each sample was above 0.97, suggesting a good repeatability among samples (Figure S2, Figure 2(a)). Cluster analysis was performed on the DEG sets in 9 samples, and genes with similar expression patterns were clustered together. These genes might have similarity functions or participate in common metabolic pathways and signal pathways. Red in the hierarchical clustering diagram indicated high expression, and blue indicated low expression (Figure 2(a)). Cluster analysis was performed on the DEG sets in 9 samples, and genes with similar expression patterns were clustered together. These genes might have similarity functions or participate in common metabolic pathways and signal pathways. Red in the hierarchical clustering diagram indicated high expression, and blue indicated low expression (Figure 2(b)).  Figure 2(c) showed there were a total of 759 DEGs (280 up- and 479 downregulated genes) in the 0-VS-1 group and 1342 DEGs (155 up- and 1187 downregulated genes) in the 0-VS-2 group.

To further analyze the differential expression induced by Cu, the top 20 DEGs of the 0-VS-1 group and 0-VS-2 group were analyzed. As shown in Figure 2(d) and Table 1, there were 3 upregulated genes and 17 downregulated genes in the 0-VS-1 group, among which *fmo-2*, *gst-33*, *cht-1*, *gst-21*, and *gst-35* were participating in metabolism, six (*hsp-70*, *hsp-16.11*, *hsp-16.2*, *hsp-16.41*, *hsp-16.48*, and *hsp-16.49*) were heat shock protein, *col-41*, *dpy-17*, and *dsl-2* were related with a component of membrane, *cav-1* was involved in reproduction, *his-66* was related to genetics, and nhr-17 had metal ion binding activity. As shown in Figure 2(d) and Table 2, there were 2 upregulated genes and 18 down-regulated genes in the 0-VS-2 group. Among them, *fat-5*, *fat-7*, *elo-7*, and icl-1 were involved in metabolism, *abu-1* and *abu-7* participated in endoplasmic reticulum stress, *adt-1*, *bli-1*, *cdh-7*, *col-17*, *col-154*, *col-157*, *dpy-4*, *dpy-8*, and *dpy-14* were structural constituent of cuticle, *adt-1* also could bind with metal ion, *fis-1* regulated mitochondrial damage, and *grd-2* was related to reproduction. The descriptions and functions of genes were shown in Tables 1 and 2.

### 3.3. Pathway Analysis of DEGs in Cu-Exposed *C. elegans*

DEGs were annotated through GO terms for gene function analysis. As showed in SI files Figure S3 A and B, there were 25 GO terms in biological process (BP), 12 GO terms in molecular function (MF), and 13 GO terms in biological process (CC) of 0-VS-1 group. As for the 0-VS-2 group, GO terms in BP, MF, and CC of were 24, 16, and 17. Compared to the control, it was obvious that DEGs and GO terms induced by Cu were much more as the exposure concentration increased.

The KEGG pathway database was used to identify differently enriched pathways of DEGs, which was contributed to explicate genes biological functions of *C. elegans* response to Cu. As shown in Figures 2(e) and 2(f), pathways mainly related to metabolism and organismal systems, including metabolism of xenobiotics by cytochrome P450, arachidonic acid metabolism, oxidative phosphorylation, glutathione metabolism, longevity regulating pathway, circadian rhythm, thermogenesis, and adipocytokine signaling pathway. Compared with the control group, 1 and 2 mg/L Cu induced analogous types of pathways but with different number of DEGs. Most pathways and DEGs were enriched in metabolism-related pathways, including fatty acid metabolism, fatty acid degradation, polyketide sugar unit biosynthesis, valine, leucine and isoleucine degradation, fatty acid biosynthesis, biosynthesis of amino acids, and cysteine and methionine metabolism. Further, top 10 pathway enrichment analyses showed most identified DEGs involved in longevity regulating pathway in the 0-VS-1 group, while most identified DEGs involved in fatty acid metabolism and degradation in the 0-VS-2 group (Figure S3 C and D). Practically, the twelfth pathway of the 0-VS-2 group was also longevity regulating pathway (Table S2).

### 3.4. Cu Induced Fatty Acid Profiling Changes of *C. elegans*

**Figure 3:**
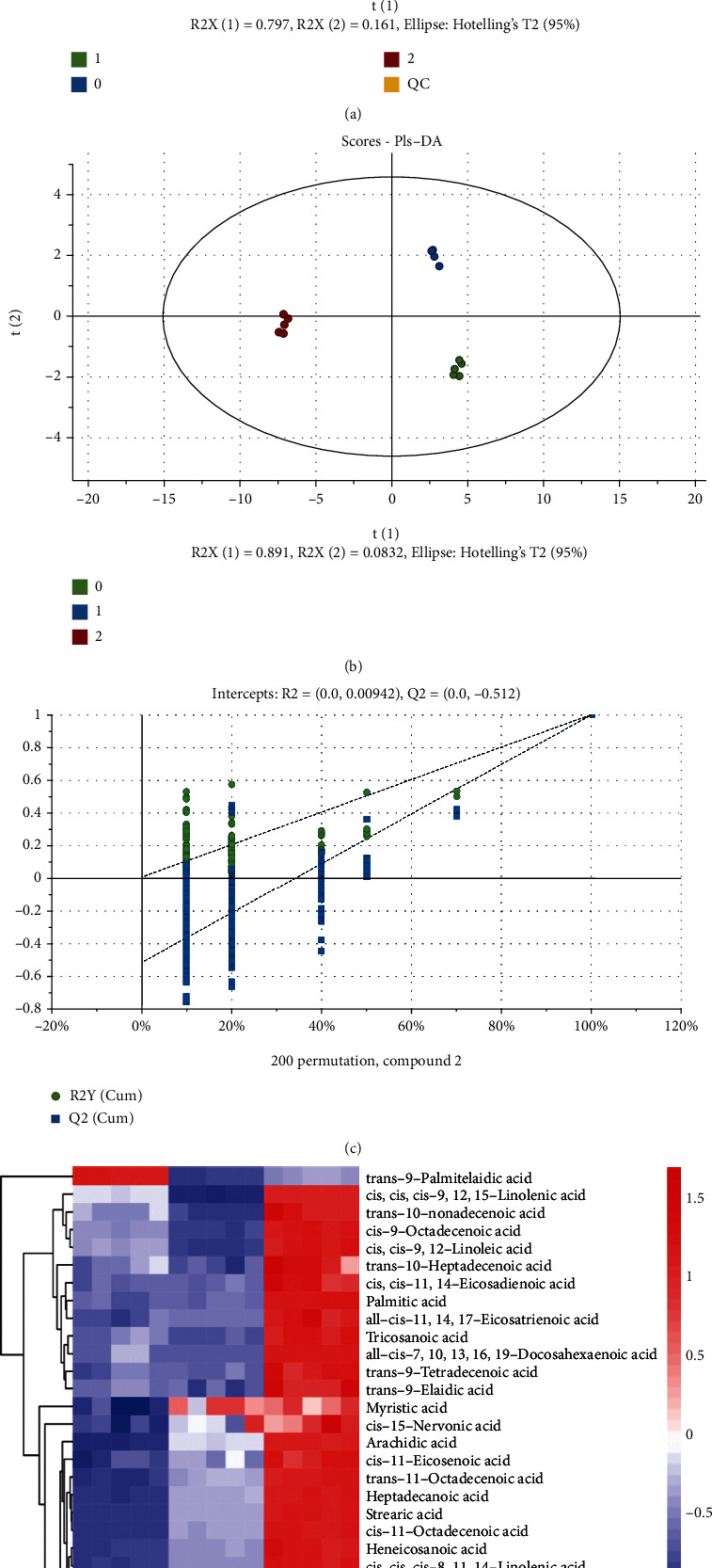
Cu induced fatty acid changes of *C. elegans.* (a) PCA plot of metabolites in Cu-exposed *C. elegans*. (b) OPLS-DA score plot of free fatty acid metabolites. (c) Plot of the permutation test of the OPLS-DA model. (d) Heatmap of the DMs generated by hierarchical clustering.

Thereafter, GC/MS metabolomic analysis was performed to investigate the effect of Cu on free fatty acid (FFA) profiling of nematodes. As shown in Figure 3(b), orthogonal partial least-squares discriminant analysis (OPLS-DA) was performed to categorize the worm samples (*R*^2^*Y* = 0.999, *Q*^2^ = 0.998). The reliability of the OPLS-DA model to explain and predict the variations was assured by model validation through permutation test (Figure 3(c)). Finally, targeted 31 FFA were analyzed, and 23 differential metabolites (DMs) of the 0-VS-1 group and 31 DMs of the 0-VS-2 group were identified (Figure 3(d), Table S4 and S5). Most of FFAs were increased, especially C14:1n5t and C18:1n7t, besides, C16:0, C16:1n7, C18:0, 18:1n9, C18:2n6, C18:3n6, C20:4n6, and C20:5n3 (EPA) were also upregulated (Supplementary Table S4 and S5). The results suggested fatty acid metabolism was disrupted by Cu.

### 3.5. Cu Exposure Promoted Fat Deposition in *C. elegans*

**Figure 4:**
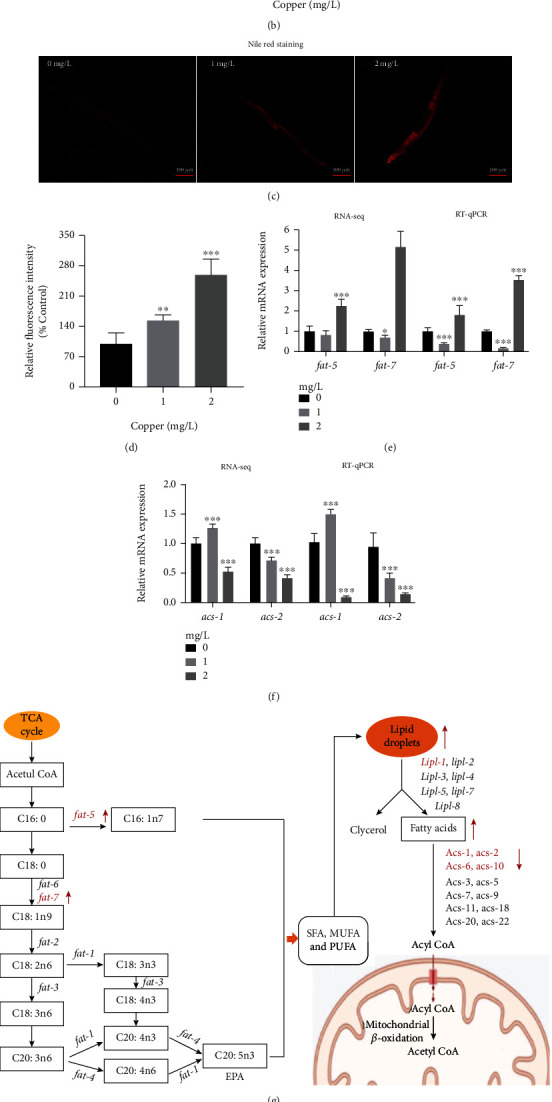
Cu exposure promoted fat deposition in *C. elegans.* (a) Representative images and (b) quantification of Oil Red O staining of N2 worms, *n* ≥ 30/group. (c) Representative images and (d) quantification of Nile Red O staining of N2 worms, *n* ≥ 30/group. (e, f) Expressions levels of lipid metabolism-related genes *fat-5*, *fat-7*, *acs-1*, and *acs-2* were detected by RNA-seq and RT-qPCR. (g) Schematic of lipid metabolism of *C. elegans* (the red arrow indicated that the gene levels were upregulated or downregulated). (h) Composition of saturated fatty acids (SFA), monounsaturated fatty acids (MUFA), and polyunsaturated fatty acids (PUFA) were analysis in Cu-treated *C. elegans*. (i) The ratio of C16:1n7/C16:0 and C18:1n9/C18:0. Scale bar = 100 *μ*m; ^∗^*P* < 0.05, ^∗∗^*P* < 0.01, and ^∗∗∗^*P* < 0.001, compared with the control. Bars represent means ± SD, *n* = 3 independent experiments.

To explore the effect of Cu exposure on fat deposition in *C. elegans*, Oil red O staining and Nile Red O staining were conducted. As shown, triglyceride (TG) or fats were obviously enhanced by Cu (Figure 4(a) and 4(b)). Lipid droplets in worms were increased in a dose-dependent manner (Figures 4(c) and 4(d)). To further explain the lipid-regulating effect of Cu, lipid metabolism-related genes of *C. elegans* were analyzed from RNA-seq results and were confirmed by RT-qPCR (Figures 4(e) and 4(f)). The *Δ*9-desaturase *fat-5* and *fat-7* gene expressions were decreased at 1 mg/L concentration and increased at 2 mg/L concentration; genes *acs-1* and *acs-2* encoding fatty acid *β*-oxidation enzymes were gradually inhibited by Cu. The polyunsaturated fatty acid (PUFA) synthesis pathway and schematic of lipid metabolism of *C. elegans* were shown in Figure 4(g), FAT-5 worked on C16:0 to synthesize C16:1n7, and FAT-6 and FAT-7 acted on C18:0 to synthesize C18:1n9 [37]. ACS enzymes, as fatty acid CoA synthetase family, were required for activating fatty acids to acyl CoA [37]. Our GC/MS results found saturated fatty acids (SFA), monounsaturated fatty acids (MUFA), and PUFA were all altered (Table S4 and 5, Figure 4(h)). To further evaluate the activity of *fat-5* and *fat-7*, product C16:1n7/precursor C16:0 ratio and product C18:1n9/precursor C18:0 ratio was compared. The C16:1n7/C16:0 ratio was increased significantly by Cu, which also verified the enhanced activity of FAT-5 (Figure 4(i)).

### 3.6. Cu Decreased MMP (*Δψ*m), Inhibited UPR^mt^, and Stimulated UPR^ER^

**Figure 5:**
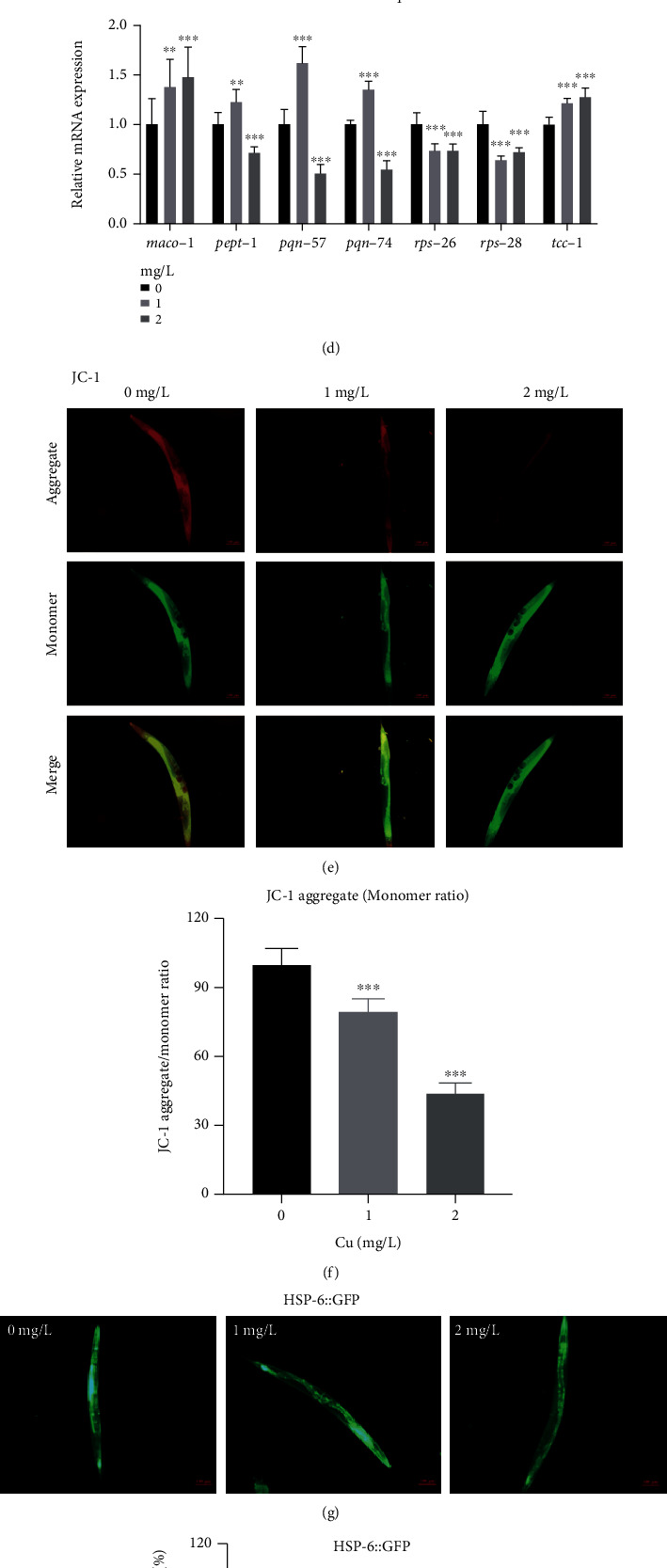
Cu stimulated UPR^ER^, decreased MMP (*Δψ*m), and inhibited UPR^mt^ of *C. elegans.* (a) Representative images of HSP-4::GFP, *n* ≥ 30/condition. (b) Quantitative of GFP fluorescence of HSP-4::GFP. (c) DEG analysis of endoplasmic reticulum-related gene regulation. (d) Gene mRNA levels of endoplasmic reticulum related genes. (e, f) The red/green fluorescence ratio of JC-1 in *C. elegans.* (g) Representative images of HSP-6::GFP, *n* ≥ 30/condition. (h) Quantitative of GFP fluorescence of HSP-6::GFP. (i) DEG analysis of mitochondrial related genes regulation. (j) Gene mRNA levels of *asg-2*, *co x-7C*, *ctc-1*, and *ctc-3.*Scale bar = 100 *μ*m; ^∗∗^*P* < 0.01, ^∗∗∗^*P* < 0.001, compared with the control. Bars represent means ± SD, *n* = 3 independent experiments.

KEGG pathway enrichment analysis suggested that impaired fatty acid degradation pathway also contributed to lipid accumulation (Figure S3 C and D). Endoplasmic reticulum was also important for fat storage and mobilization [38]. We sought to determine the effect of Cu on UPR^ER^ of worms. As shown, reporter HSP-4::GFP was inhibited at first and then was upregulated (Figures 5(a) and 5(b)). In addition, DEGs associated to the endoplasmic reticulum were analyzed. There were 66 DEGs in the 0-VS-1 group and 74 DEGs in 0-VS-2 group, and 15 overlap of genes (*abu-6*, *abu-7*, *abu-8*; *hsp-16.11*, *hsp-16.41*, *hsp-16.48*, *hsp-16.49*, *hsp-70*; *maco-1*, *pept-1*; *pqn-57*, *-74*; rps-26, rps*-28*; and *tcc-1*, Figure 5(c)). The mRNA expressions and descriptions of the 15 genes were showed in Figure 5(d) and Table S2.

Mitochondrial dysfunction impaired lipid homeostasis. Thus, mitochondrial membrane potential (MMP, *Δψ*m) and mitochondrial unfolded protein response (UPR^mt^) were detected. The result of JC-1 probes staining showed that J-aggregates (red fluorescence)/monomeric (green fluorescence) ratios were reduced with Cu treatment (Figures 5(e) and 5(f)), revealing a decreased *Δψ*m. The expression of HSP-6::GFP presented decrease trend (Figures 5(g) and 5(h)). Further, we analyzed DEGs induced by Cu associated to mitochondrial. There were 86 DEGs in the 0-VS-1 group and 31 DEGs in 0-VS-2 group and 4 overlap of genes (*asg-2*, *cox-7C*, *ctc-1*, and *ctc-3*, Figure 5(i)). Figures 5(i) and 5(j) and Table S2 showed mRNA expression and biological functions of the above 4 genes.

### 3.7. DAF-16 Mediated Cu-Induced Aging and Oxidative Stress

**Figure 6:**
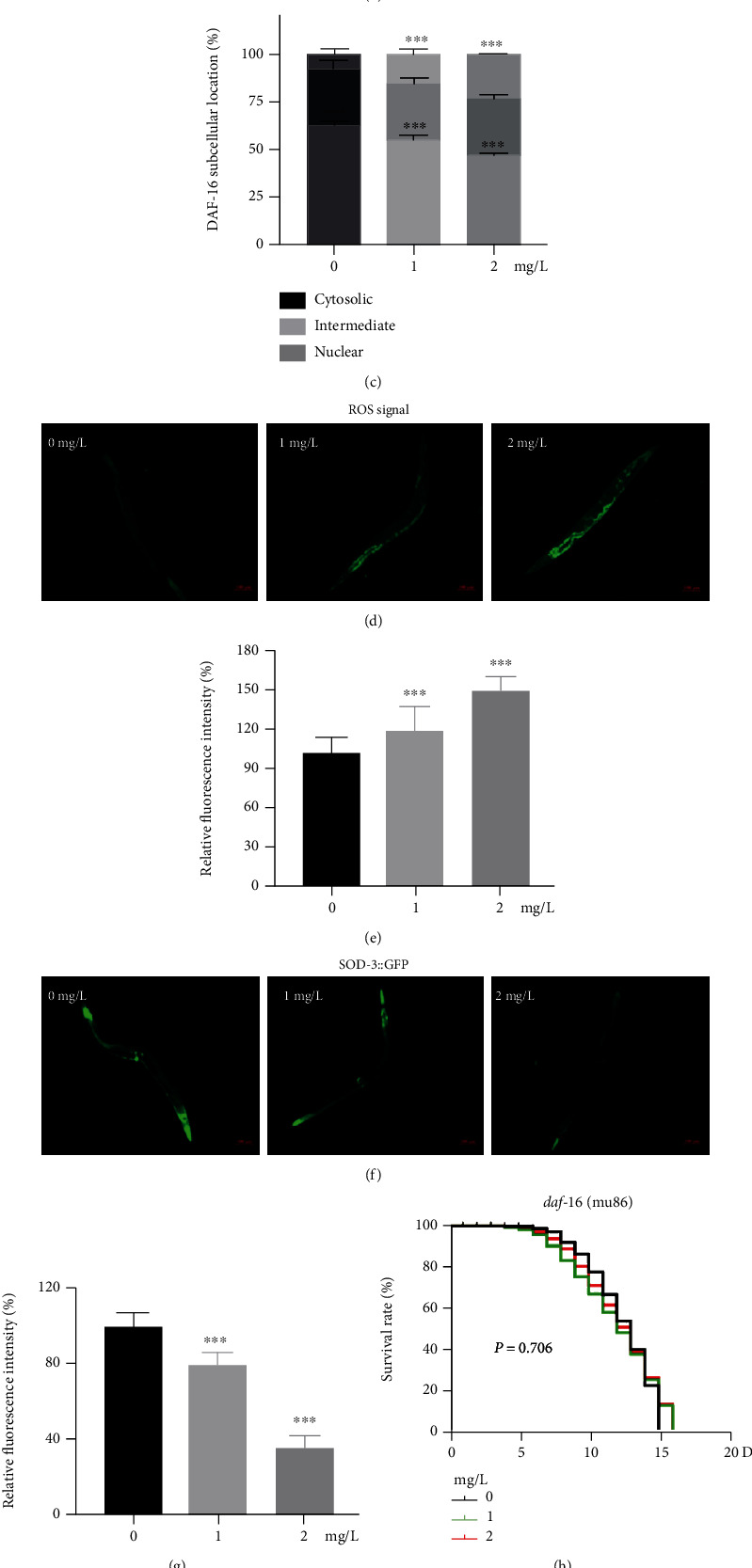
DAF-16 mediated Cu-induced aging and oxidative stress. (a) Expressions levels of IIS pathway related genes *daf-2*, *age-1*, and *daf-16* were detected by RNA-seq and RT-qPCR. (b) Images of daf-16-GFP fusion gene in TJ356 worms, *n* ≥ 30/group. (c) Quantitative of DAF-16 localization. (d) H2DCFDA probe was performed to detect ROS level, *n* ≥ 30/condition. (e) Quantitative analysis of fluorescence intensities. (f) Representative images of SOD-3::GFP, *n* ≥ 30/condition. (g) Quantitative of GFP fluorescence in SOD-3::GFP worms, *n* ≥ 30/condition. (h) Kaplan-Meier survival curve of *daf-16* (mu86) strain, 30 nematodes/plate, total 360 worms/condition. (i) mRNA level of SOD-related genes and metal detoxification genes. Scale bar is 100 *μ*m, ^∗^*P* < 0.05, ^∗∗^*P* < 0.01, and ^∗∗∗^*P* < 0.001, compared with the control. Bars represent means ± SD, *n* = 3 independent experiments.

IIS pathway was related to regulation of longevity, aging, and fat metabolism. Results of RNA-seq and RT-qPCR revealed that *daf-2* and *age-1* were promoted (Figure 6(a)) and downstream genes of *daf-16* were inhibited by Cu; however, the expression of *daf-16* was not changed significantly. Therefore, we speculated whether Cu prevented the daf-16 *trans*-location to the nucleus through insulin signaling to regulate fat storage and lifespan. DAF-16 was mainly in the cytosolic of worm cells, for inducing nuclear localization of DAF-16, TJ356 worms were starved for 16 h. Then, Cu exposure altered the DAF-16 localization. The result showed (Figures 6(b) and 6(c)) that DAF-16 translocated out of the nucleus, indicating DAF-16 translocation to the nucleus was inhibited by Cu. DAF-16 directly regulated the expression of a variety of genes, such as *sod-3*, *gst-4, ctl-2*, and *mtl-1*, which are involved in oxidative stress [39]. The H2DCFDA probe staining results showed that ROS level was increased with Cu treatment (Figures 6(d) and 6(e)). Further, CF1553 (SOD-3::GFP) reporter strain was used to explore the effects of Cu on the antioxidant enzyme. The result showed the fluorescence levels of SOD-3::GFP were decreased by Cu (Figures 6(f) and 6(g)). To further elucidate the regulation of DAF-16 on lifespan, *daf-16* (mu86) strain was used to evaluate survival after Cu treatment. However, Cu did not alter the lifespan of the mutant strain significantly compared with untreated worm (Figure 6(h)). In addition, RNA-seq results revealed that mRNA level of *sod-1*, *sod-2*, *sod-3*, *mlt-1*, and *mlt-2* were inhibited by Cu (Figure 6(i)), the specific FC value and description of the genes were shown in Table S2 of SI.

## 4. Discussion

The early-life period is an important window of susceptibility to environmental exposures. Different developmental stages would be critical windows of exposure. Developmental exposure to toxicants may result in an acceleration of age-related decline in function. A study showed different exposure paradigms (i.e., L1 stage treatment or L4 stage treatment) induced differentially expressed genes (DEGs) [40]. Therefore, we conducted two different exposure paradigms of Cu (L1 stage and L4 stage of *C. elegans*) during RNA sequencing process. Interestingly, Cu exposure promotes aging in *C. elegans* at different stages, but the involving different mechanisms. Our previous results show that Cu induced 2332 DEGs (567 up- and 1765 downregulated genes) of the 1 mg/L group and 2449 DEGs (724 up- and 1725 downregulated genes) of 2 mg/L group in the L1 stage exposure paradigm [31]. In this study, Cu induced 759 DEGs (280 up- and 479 downregulated genes) of the 1 mg/L group and 1342 DEGs (155 up- and 1187 downregulated genes) of the 2 mg/L group in L4 stage exposure paradigm. Further, the top 20 DEGs were analyzed.

In the L1 stage exposure paradigm, the top 20 DEGs were involved in structural constituent of cuticle or integral component of membrane, immune response, lipid transport, epigenetic regulation, and gene expression regulation [31]. However, in the L4 stage exposure paradigm, the top 20 DEGs were participating in component of membrane, metabolism, heat shock stress, endoplasmic reticulum stress, and mitochondrial damage. Further, identification of the pathway showed two concentrations in the L1 stage exposure paradigm both influenced on the lysosome, longevity regulating, and peroxisome pathway [31]. In the L4 stage exposure paradigm, two concentrations both induced longevity regulating pathway and adherens junction. Interestingly, Cu mainly affected the lipid metabolism with increasing concentrations. Therefore, the direction of the mechanism verification of the two Cu exposure paradigms is also different. Cu exposure widely affects longevity regulation pathways in the L1 stage exposure paradigm, thereby promoting aging [31]. In the L4 stage exposure paradigm, we focus on fat metabolism, mitochondrial dysfunction, endoplasmic reticulum emergency, and oxidative stress according to clues of RNA sequencing.

This study is aimed at exploring the health damage after L4 stage *C. elegans* exposed to Cu for 24 h. Transcriptome sequencing and quantitative detection of free fatty acid of Cu-treated nematodes were conducted to analyze the molecular mechanism of Cu-induced abnormal behavior and function of nematodes.

### 4.1. Cu Promoted Aging and Behavioral Disorders of *C. elegans*

Aging composites the important risk factor for chronic degenerative diseases, such as cancer and cardiovascular [41]. It was very important to explore the occurrence and mechanism of aging. *C. elegans* was an excellent model to study with aging due to its physical decline strikingly similar to humans [42]. Typical markers of aging-relative phenotype in *C. elegans* included the locomotion decline, feeding ability (pharyngeal pumping frequency), defecation ability (defecation interval), morphological changes, and the aging-associated genes [29, 43].

First of all, lifespan was shortened by two environmentally relevant doses of Cu. Body length and width were used to evaluate the development of *C. elegans*, the result of declined body length and body width suggested that *C. elegan*s was sensitivity to growth inhibition by Cu. Pharyngeal pumping was controlled by corpus and isthmus muscles, which was regulated by neurons and muscle-intrinsic molecules [44]. A healthy well-fed adults *C. elegans* pumped 200-300 times per minute [29, 44]. Defecation was a kind of rhythmic exercise that consists a three-part motor program coordinated by neural activity [29]. A healthy well-fed young adult wild-type *C. elegans* defecated once every 45 seconds [29]. Both pharyngeal pumping frequency and defecation interval were correlated with the lifespan.

In the study, the pharyngeal pumping rate was decreased by Cu, the defecation interval was prolonged. The above research results preliminary indicated that Cu decreased the lifespan, impaired development and promoted aging-related behaviors of *C. elegans.*

To illustrate the regulatory mechanism behind these recessions induced by Cu, RNA-seq was performed. Transcriptomics data showed cuticle structure related genes included cuticle collagen *bli-1*; collagen *col-17*, *col-41, col*-154 and *col-157*; and collagen structural *dpy-4*, *dpy-8*, *dpy-14,* and *dpy-17* were downregulated; besides, other 54 collagen-encoded genes were also suppressed including *col-8*, *col-14*, and *col-39*. *col-8*, *col-14*, and *col-39* belonged to *C. elegans* cuticle collagen cysteine families [45]. These genes encoded external structure, that is, cuticle, and were crucial for development and survival of nematode [46, 47], which well explained that Cu inhibited their development. Behavior and lifespan were affected by several pathways, including the inositol phosphate pathway, lipid metabolism pathways, and energy metabolism pathways. Therefore, we would conduct an in-depth discussion in conjunction with subsequent experiments.

### 4.2. Cu Might Regulate Fat Metabolism via UPR^mt^ and UPR^ER^

The effect on metabolism of *C. elegans* seemed to be more pronounced with Cu exposure increases. Analysis of DGEs, GO function annotation, and KEGG pathway were all pointing that abnormal lipid metabolism was produced by Cu. DGEs were involved in fatty acid degradation, metabolism, and biosynthesis. Lipids executed many essential functions, for example, they were efficient energy storage molecules, formed a double-layer membrane structure, and played as signaling molecules [48]. However, incorrectly lipid homeostasis would develop dysregulated neurological and developmental consequences [37]; moreover, lipid overload could reduce lifespan of *C. elegans* [37, 49].

Lipid metabolism was set of process that comprised lipid synthesis at endoplasmic reticulum, storage and transport of lipid droplets, *β*-oxidation at mitochondria and peroxisomes, and lipid hydrolysis and recycling at lysosomes [50–52]. Firstly, we found 2 mg/L Cu upregulated delta (9)-fatty-acid desaturase (∆9 desaturase) *fat-5* and *fat-7*. ∆9 desaturase was considered to be lipogenic enzymes and active in endoplasmic reticulum membrane both in mammals and *C. elegans* [53, 54]. FAT-5 worked on C16:0 to produce C16:1n-7, and FAT-7 mainly acted on C18:0 to produce C18:1n-9 [55]. Thus, we conducted targeted FFA metabolomic analysis in *C. elegans* and found FFA was increased after Cu treated. Correspondingly, C16:0, C16:1n-7, C18:0, and C18:1n-9 were all increased. Oil red staining and Nile red staining further confirmed that Cu promoted fat accumulation in N2 nematodes. Further, we found Cu induced ER stress of *C. elegans*, and multiple proteins associated with endoplasmic reticulum were affected by Cu. The ER was to facilitate the folding and maturation of protein molecules [56, 57]. AS reported that misfolded proteins would be activated to cope with ER stress [56]. Thus, Cu stimulated ER stress on the one hand to activate expression of *fat-5* and *fat-7*, and on the other hand, to increase the expression of HSP-4::GFP sequestrated to the misfolded protein.

The end products of lipid mobilization, FFAs, went to further degradation via mitochondrial *β*-oxidation, which was related to longevity mechanisms [58]. In the *C. elegans* intestine, *asc-1* and *acs-2* encoded acyl-CoA synthetases (ACSs), which could activate FFAs *β*-oxidation at mitochondrial [59, 60]. However, *asc-1* and *acs-2* were downregulated in our study. To explore the mechanism behind, it was found that MMP (*Δψ*m) and UPR^mt^ were both reduced in Cu-treated N_2_ worms. The loss of MMP (*Δψ*m) was a hallmark of mitochondrial dysfunction [61]. Mitochondria was the core organelles for energy metabolism, programmed cell death, and metabolic pathways involving lipids and amino acids [62]. Environmental stress or dysfunction led to accumulation of unfolded protein would trigger UPR^mt^, which was a compensatory mechanism to maintain mitochondrial homeostasis [63, 64]. Surprisingly, we found that a series of ribosomal proteins located in the mitochondrial were dysfunctional, and some of them were firstly downexpressed then overexpressed as Cu exposure increasing (SI Figure S4). The influence of the mitochondrial ribosomal proteins (MRPs) on lifespan was conserved from mammals to *C. elegans* that triggers the UPR^mt^ by downexpression of MRPs was a life-extending mechanism [65]. On the contrary, suppressed UPR^mt^ contributed to aging.

Phenotype is the result of a combination of inheritance and gene regulation and involves a myriad of changes at the organ, tissue, and cellular levels. Inactivation of C. elegans desaturase and elongase family members, encoded by fat and elo genes, respectively, causes imbalances in fatty acid composition and is associated with metabolic, physiological, and behavioral phenotypes [66]. These include altered total fat levels, growth retardation, slowed movement, reduction in body size, germ cell maintenance and reproductive defects, aberrations in rhythmic behavior, defects in sensory signaling, defects in neurotransmission, and reduced adult lifespan [37]. Similarly, lipid storage phenotypes in this study was a comprehensive result of Cu-induced oxidative damage, mitochondrial dysfunction, and endoplasmic reticulum stress, even though the gene expression changes of lipid metabolism genes vary. Thus, the exact mechanism of lipid storage induced by Cu was still need discussed in depth.

### 4.3. Cu Prevented *daf-16* from Activating Downstream Genes through Oxidation Damage

Aging was accompanied by functional and structural declines. It was interesting that most of the DEGs were downregulated, indicating Cu might play a role of inhibitors during exposure. Focus on the top20 DEGs induced by 1 mg/L Cu, six downregulated heat shock protein *hsp-16.1/16.11*, *hsp-16.2*, *hsp-16.41*, *hsp-16.48*, *hsp-16.49*, and *hsp-70* were related to aging. HSP-16.1, HSP-16.2, HSP-16.41, and HSP-16.48 could protect *C. elegans* to environmental stress, such as heat shock, ROS, beta-amyloid peptide, heavy metals, and nanomaterials [67–69]. HSPs were regulated by DAF-16 and participating in the longevity regulating pathway [70, 71]. DAF-16/FOXO transcription factor, controlled by DAF-2/IGFR activating phosphoinositide 3-kinase AGE-1/PI3K kinase cascade, governed most of the functions of the insulin/IGF-1 signaling (IIS) pathway [72]. Activated DAF-2 receptor could recruit and activate AGE-1, resulting in phosphorylation of and cytoplasmic sequestration of the DAF-16. The *C. elegans* IIS pathway extensively regulated metabolism, growth, development, behavior, and longevity [73, 74].

To further confirm the effect of Cu on IIS pathway, genes (*daf-2*, *age-1*, and *daf-16*) based on the RNA-seq data were selected for RT-qPCR validation. It showed that *daf-2* and *age-1* were activated, and image analysis of TJ356 suggested DAF-16 nuclear localization was decreased. Next, glutathione S-transferase, *gst-21* and *gst-33* were found downregulated, and *gst-35* was upregulated, which were a response to oxidative stress [75]. Accordingly, we further confirmed that Cu caused accumulation of ROS accompanied by decreased expression of antioxidant SOD-3. As shown in Table S2, *C. elegans* had five SOD genes that *sod-1* and *sod-5* encoded cytoplasmic CuZnSOD, *sod-4* encoded extracellular CuZnSOD, and *sod-2* and *sod-3* encoded mitochondrial MnSOD. *sod-5* was stimulant increased while another *sod-1* to *sod-4* were inhibited, especially *sod-1* and *sod-2*. Two other genes that could not be ignored, *mtl-1* and *mtl-2*, that encoded metallothionein were involved in stress response to heavy metal in *C. elegans* [76]. As reported, ROS served as pleiotropic physiological signaling agents which could also stimulate DAF-2/IGFR signaling [77, 78]. Additionally, *sod-3, mtl-1*, and *mtl-2* were direct transcript targets of DAF-16 and were related to longevity [79, 80].

Taken together, Cu exposure activated ROS; then, oxidative damage signals directly or indirectly sequestrated DAF-16 in the cytoplasm, contributing to the DAf-16 fail of regulation target gene transcription (such as *sod-3*, MTLs and HSPs), finally decreased stress resistance of *C. elegans*.

## 5. Conclusion

In summary, our study implicated Cu accelerated *C. elegans* aging accompanied by developmental and behavioral suppression. Further mechanism study showed Cu induced fat deposition and oxidation damage. Cu, on the one hand, triggered UPRER which led to the upregulation of ∆9 desaturase FAT-5 and FAT-7 to increase lipid synthesis, and on the other hand, inhibited MMP and induced an imbalance of MRP, thereby inhibiting UPR^mt^ and FFAs *β*-oxidation. In addition, Cu activated ROS and at least in part prevented antioxidant capacity through transcription factor DAF-16/FOXO.

The study illustrated the potential threats of Cu to humans at part degree and provided some theoretical basis for preventing or mitigating aging induced by Cu.

## Figures and Tables

**Table 1:** Top 20 significantly DEGs of *C. elegans* in response to 1 mg/L Cu.

	Symbol	Description	Function	Log_2_FC^∗^
Up	*fmo-2*	Dimethylaniline monooxygenase	N,N-Dimethylaniline monooxygenase activity, NAD(P)H oxidase, H_2_O_2_-forming activity, NADP binding, flavin adenine dinucleotide binding, monooxygenase activity	1.33
	*bath-15*	BTB and MATH domain-containing protein 15	SKN-1-dependent zygotic transcript	1.14
	*gst-33*	Glutathione S-transferase	Metabolism of xenobiotics by cytochrome P450	1.01
Down	*cav-1*	Caveolin-1	Ras protein signal transduction, meiotic cell cycle, regulation of oviposition, transport vesicle	-1.55
	*cht-1*	Putative endochitinase	Chitinase activity, hydrolase activity, acting on glycosyl bonds, carbohydrate metabolic process, chitin catabolic process, polysaccharide catabolic process	-1.11
	*col-41*	COLlagen	Structural constituent of cuticle	-1.22
	*dpy-17*	Col_cuticle_N domain-containing protein	The dpy-17 encodes a collagen known to genetically interact with dpy-31, a BMP-1/Tolloid-like metalloprotease required for TGFbeta activation in mammals	-1.42
	*dsl-2*	Delta-like protein	Cell fate specification, integral component of plasma membrane	-1.28
	*his-66*	Putative histone H2B 4	DNA binding, protein heterodimerization activity	-3.79
	*nhr-17*	Nuclear hormone receptor family	Metal ion binding, cell differentiation, anatomical structure development, intracellular receptor signaling pathway, regulation of transcription by RNA polymerase II, DNA-templated	-3.79
	*gst-21*	Glutathione S-transferase	Glutathione transferase activity, glutathione metabolic process	-2.61
	*gst-35*			-2.30
	*hsp-70*	Heat shock protein	IRE1-mediated unfolded protein response, cellular response to unfolded protein, chaperone cofactor-dependent protein refolding, determination of adult lifespan IGI, endoplasmic reticulum unfolded protein response	-1.62
	*hsp-16.11*	Heat shock protein-16.1/Hsp-16.11	Unfolded protein binding, IRE1-mediated unfolded protein response, endoplasmic reticulum unfolded protein response	-1.98
	*hsp-16.2*	Heat shock protein-16.2		-2.39
	*hsp-16.41*	Heat shock protein-16.41		-2.47
	*hsp-16.48*	Heat shock protein-16.48	IRE1-mediated unfolded protein response, determination of adult lifespan, endoplasmic reticulum unfolded protein response, response to heat	-1.88
	*hsp-16.49*	Heat shock protein-16.49		-1.88
	—	Regulator of rDNA transcription protein 15	—	-2.13
	—	Unidentified protein	—	-1.04

^∗^FC: fold change.

**Table 2:** Top 20 significantly DEGs of *C. elegans* in response to 2 mg/L Cu.

	Symbol	Description	Function	Log_2_FC^∗^
Up	*fat-5*	Delta (9)-fatty-acid desaturase fat-5	Oxidoreductase activity, acting on paired donors, with oxidation of a pair of donors resulting in the reduction of molecular oxygen to two molecules of water, stearoyl-CoA 9-desaturase activity fatty acid biosynthetic process	1.14
	*fat-7*	Delta (9)-fatty-acid desaturase fat-7		2.38
Down	abu-1	Activated in blocked unfolded protein response	Endoplasmic reticulum unfolded protein response, integral component of endoplasmic reticulum membrane	-1.14
	*abu-7*		Endoplasmic reticulum unfolded protein response, pharynx development	-1.07
	*adt-1*	A disintegrin and metalloproteinase with thrombospondin motifs *adt-1*	Hydrolase activity, metal ion binding, metalloendopeptidase activity, extracellular matrix organization, proteolysis, extracellular matrix	-1.31
	*bli-1*	Cuticle collagen *bli-1*	Extracellular matrix structural constituent, structural constituent of collagen and cuticulin-based cuticle, cuticle development involved in collagen and cuticulin-based cuticle molting cycle	-1.41
	*cdh-7*	CaDHerin family	Calcium ion binding, homophilic cell adhesion via plasma membrane adhesion molecules, integral component of membrane	-1.19
	*col-17*	Collagen	Collagen trimer	-1.02
	*col-154*	Col_cuticle_N domain-containing protein	Structural constituent of cuticle, integral component of membrane	-1.17
	*col-157*			-1.17
	*dpy-4*		Extracellular matrix structural constituent, structural constituent of cuticle, cuticle development involved in collagen and cuticulin-based cuticle molting cycle	-1.09
	*dpy-8*			-1.09
	*dpy-14*			-1.95
	*elo-7*	Elongation of very long chain fatty acids protein	Activity of 3-oxo-arachidoyl-CoA synthase, 3-oxo-cerotoyl-CoA synthase, 3-oxo-lignoceronyl-CoA synthase and very-long-chain 3-ketoacyl-CoA synthase	-1.04
	*fis-1*	Mitochondrial fission 1 protein	Fis1 can act in sequence with Mff at the ER-mitochondrial interface to couple stress-induced mitochondrial fission.	-1.10
	*grd-2*	GRounDhog (hedgehog-like family)	Cholesterol binding, endopeptidase activity, signaling receptor binding, cell-cell signaling, protein auto-processing	-1.43
	*icl-1*	Malate synthase	Catalytic activity, isocitrate lyase activity, lyase activity, malate synthase activity, carboxylic acid metabolic process, determination of adult lifespan IGI, glyoxylate cycle, tricarboxylic acid cycle	-1.36
	*H17B01.2*	Uncharacterized protein		-1.47
	—	Protein CBG06623		-1.32
	—	C-type LECtin		-1.12

^∗^FC: fold change.

## Data Availability

All data included in this study were available upon request by contact with the corresponding author.

## Supplementary Materials

Supplementary Materials (1) Sample preparation, library construction, and RNA sequencing. (2) Bioinformatics analysis. (3) Detection of ROS and Mitochondrial membrane potential (MMP, *Δψ*m). (4) Metabolite extraction and LC-MS/MS analysis. Figure S1: violin plot for all samples. Figure S2: correlation analysis for all samples. The correlation of each sample was above 0.97. Figure S3: GO enrichment analysis and top 10 pathways enrichment. Figure S4: mitochondrial ribosomal proteins were transcriptionally repressed by Cu. Table S1: the gene primer used in the study. Table S2: expression of differentially expressed genes (DEGs) in copper-exposed *C. elegans*. Table S3: top 12 statistics of pathways enrichment. Table S4: differential free fatty acid induced by 1 mg/L copper. Table S5: differential free fatty acid induced by 2 mg/L copper.

## References

[1] Chen B., Han M. Y., Peng K. (2018). Global land-water nexus: agricultural land and freshwater use embodied in worldwide supply chains. *Science of the Total Environment*.

[2] Weerasooriya R. R., Liyanage L. P. K., Rathnappriya R. H. K. (2021). Industrial water conservation by water footprint and sustainable development goals: a review. *Environment Development and Sustainability*.

[3] Xia X. H., Chen X., Liu R. M., Liu H. (2011). Heavy metals in urban soils with various types of land use in Beijing, China. *Journal of Hazardous Materials*.

[4] Oubane M., Khadra A., Ezzariai A., Kouisni L., Hafidi M. (2021). Heavy metal accumulation and genotoxic effect of long-term wastewater irrigated peri-urban agricultural soils in semiarid climate. *Science of the Total Environment*.

[5] White L., Petrovitch H., Ross G. W. (1996). Prevalence of dementia in older Japanese-American men in Hawaii: the Honolulu-Asia Aging Study. *JAMA*.

[6] Zoroddu M. A., Aaseth J., Crisponi G., Medici S., Peana M., Nurchi V. M. (2019). The essential metals for humans: a brief overview. *Journal of Inorganic Biochemistry*.

[7] Bost M., Houdart S., Oberli M., Kalonji E., Huneau J. F., Margaritis I. (2016). Dietary copper and human health: current evidence and unresolved issues. *Journal of Trace Elements in Medicine and Biology*.

[8] Andra S. S., Makris K. C., Charisiadis P., Costa C. N. (2014). Co-occurrence profiles of trace elements in potable water systems: a case study. *Environmental Monitoring and Assessment*.

[9] Vargas I. T., Fischer D. A., Alsina M. A., Pavissich J., Pastén P., Pizarro G. (2017). Copper corrosion and biocorrosion events in premise plumbing. *Materials (Basel)*.

[10] Eife R., Mullerhocker J., Weiss M. (1989). Familial’ infantile liver cirrhosis and hemolytic anemia due to chronic copper intoxication via tap water from copper pipes. *Pediatric Research*.

[11] Monterroso C., Rodriguez F., Chaves R. (2014). Heavy metal distribution in mine-soils and plants growing in a Pb/Zn-mining area in NW Spain. *Applied Geochemistry*.

[12] Morris M. C., Evans D. A., Tangney C. C. (2006). Dietary copper and high saturated and trans fat intakes associated with cognitive decline. *Archives of Neurology*.

[13] Kronzucker H. J., Coskun D., Schulze L. M., Wong J. R., Britto D. T. (2013). Sodium as nutrient and toxicant. *Plant and Soil*.

[14] Jin J., Mulesa L., Carrilero R. M. (2017). Trace elements in parenteral nutrition: considerations for the prescribing clinician. *Nutrients*.

[15] Medeiros D. M. (2016). Copper, iron, and selenium dietary deficiencies negatively impact skeletal integrity: a review. *Experimental Biology and Medicine (Maywood, N.J.)*.

[16] Scheiber I., Dringen R., Mercer J. F. (2013). Copper: effects of deficiency and overload. *Metal Ions in Life Sciences*.

[17] Wierzbicka D., Gromadzka G. (2014). Ceruloplasmin, hephaestin and zyklopen: the three multicopper oxidases important for human iron metabolism. *Postȩpy Higieny i Medycyny Doświadczalnej (Online)*.

[18] Letelier M. E., Faúndez M., Jara-Sandoval J. (2009). Mechanisms underlying the inhibition of the cytochrome P450 system by copper ions. *Journal of Applied Toxicology*.

[19] Bonaccorsi M., Knight M. J., Le Marchand T. (2020). Multimodal response to copper binding in superoxide dismutase dynamics. *Journal of the American Chemical Society*.

[20] Tong K. Y., Zhao J., Tse C. W., Wan P. K., Rong J., Au-Yeung H. Y. (2019). Selective catecholamine detection in living cells by a copper-mediated oxidative bond cleavage. *Chemical Science*.

[21] Agency U S E P (2019). *EPA’s lead and copper rule proposal-EPA is proposing the first major overhaul of the lead and copper rule (LCR) since 1991*.

[22] Brown R. A., McTigue N. E., Cornwell D. A. (2013). Strategies for assessing optimized corrosion control treatment of lead and copper. *Journal American Water Works Association*.

[23] Corsi A. K., Wightman B., Chalfie M. (2015). A transparent window into biology: a primer on *Caenorhabditis elegans* (vol 200, pg 387, 2015). *Genetics*.

[24] Hunt P. R. (2017). The *C. elegans* model in toxicity testing. *Journal of Applied Toxicology*.

[25] Leung M. C. K., Williams P. L., Benedetto A. (2008). *Caenorhabditis elegans*: An emerging model in biomedical and environmental toxicology. *Toxicological Sciences*.

[26] Stiernagle T. (2007). Maintenance of *C. elegans*. *Worm Book*.

[27] Li Z., Song L. (2012). Interpretation of the guidelines for drinking-water quality (fourth edition) issued by World Health Organization. *Water & Wastewater Engineering*.

[28] Organization W. H. (2017). *Guidelines for drinking-water quality, in Incorporating the 1st Addendum*.

[29] Collins J. J., Huang C., Hughes S., Kornfeld K. (2008). The measurement and analysis of age-related changes in *Caenorhabditis elegans*. *Worm Book*.

[30] Ju J. J., Saul N., Kochan C. (2014). Cyanobacterial xenobiotics as evaluated by a *Caenorhabditis elegans* neurotoxicity screening test. *International Journal of Environmental Research and Public Health*.

[31] Zhang Y., Zhao C., Zhang H. (2021). Integrating transcriptomics and behavior tests reveals how the *C. elegans* responds to copper induced aging. *Ecotoxicology and Environmental Safety*.

[32] Love M. I., Huber W., Anders S. (2014). Moderated estimation of fold change and dispersion for RNA-seq data with DESeq2. *Genome Biology*.

[33] Liu H., Tian L., Wang S., Wang D. (2021). Size-dependent transgenerational toxicity induced by nanoplastics in nematode *Caenorhabditis elegans*. *Science of the Total Environment*.

[34] Walter P. L., Kampkotter A., Eckers A. (2006). Modulation of FoxO signaling in human hepatoma cells by exposure to copper or zinc ions. *Archives of Biochemistry and Biophysics*.

[35] Dunn W. B., Broadhurst D., Begley P. (2011). Procedures for large-scale metabolic profiling of serum and plasma using gas chromatography and liquid chromatography coupled to mass spectrometry. *Nature Protocols*.

[36] Chen S. F., Zhou Y. Q., Chen Y. R., Gu J. (2018). fastp: an ultra-fast all-in-one FASTQ preprocessor. *Bioinformatics*.

[37] Watts J. L., Ristow M. (2017). Lipid and carbohydrate metabolism in *Caenorhabditis elegans*. *Genetics*.

[38] Daniele J. R., Higuchi-Sanabria R., Durieux J. (2020). UPR^ER^ promotes lipophagy independent of chaperones to extend life span. *Science Advances*.

[39] Zhang P., Judy M., Lee S. J., Kenyon C. (2013). Direct and indirect gene regulation by a life-extending FOXO Protein in *C. elegans*: roles for GATA factors and lipid gene regulators. *Cell Metabolism*.

[40] Helmcke K. J., Aschner M. (2010). Hormetic effect of methylmercury on *Caenorhabditis elegans*. *Toxicology and Applied Pharmacology*.

[41] Lopez-Otin C., Blasco M. A., Partridge L., Serrano M., Kroemer G. (2013). The hallmarks of aging. *Cell*.

[42] Lee G.-Y., Sohn J., Lee S.-J. V. (2021). Combinatorial approach using *Caenorhabditis elegans* and mammalian systems for aging research. *Molecules and Cells*.

[43] Golden T. R., Melov S. (2007). Gene expression changes associated with aging in *C. elegans*. *Worm Book*.

[44] Avery L., You Y. J. (2012). *C. elegans* feeding. *Worm Book*.

[45] Kramer J. M. (1994). Structures and functions of collagens in *Caenorhabditis elegans*. *The FASEB Journal*.

[46] Johnstone I. L. (1994). The cuticle of the nematode *Caenorhabditis elegans*: a complex collagen structure. *BioEssays*.

[47] Page A. P., Stepek G., Winter A. D., Pertab D. (2014). Enzymology of the nematode cuticle: a potential drug target?. *International Journal for Parasitology-Drugs and Drug Resistance*.

[48] Shevchenko A., Simons K. (2010). Lipidomics: coming to grips with lipid diversity. *Nature Reviews. Molecular Cell Biology*.

[49] Franco-Juárez B., Gómez-Manzo S., Hernández-Ochoa B. (2021). Effects of high dietary carbohydrate and lipid intake on the lifespan of *C. elegans*. *Cell*.

[50] Phillips M. J., Voeltz G. K. (2016). Structure and function of ER membrane contact sites with other organelles. *Nature Reviews. Molecular Cell Biology*.

[51] Shai N., Schuldiner M., Zalckvar E. (2016). No peroxisome is an island -- peroxisome contact sites. *Biochimica et Biophysica Acta-Molecular Cell Research*.

[52] Tatsuta T., Scharwey M., Langer T. (2014). Mitochondrial lipid trafficking. *Trends in Cell Biology*.

[53] Sampath H., Ntambi J. M. (2011). The role of stearoyl-CoA desaturase in obesity, insulin resistance, and inflammation. *Annals of the New York Academy of Sciences*.

[54] Brock T. J., Browse J., Watts J. L. (2007). Fatty acid desaturation and the regulation of adiposity in *Caenorhabditis elegans*. *Genetics*.

[55] Watts J. L., Browse J. (2000). A palmitoyl-CoA-specific *Δ*9 fatty acid desaturase from *Caenorhabditis elegans*. *Biochemical and Biophysical Research Communications*.

[56] Hetz C., Chevet E., Oakes S. A. (2015). Proteostasis control by the unfolded protein response. *Nature Cell Biology*.

[57] Smith H. L., Mallucci G. R. (2016). The unfolded protein response: mechanisms and therapy of neurodegeneration. *Brain*.

[58] Ratnappan R., Amrit F. R., Chen S. W. (2014). Germline signals deploy NHR-49 to modulate fatty-acid *β*-oxidation and desaturation in somatic tissues of *C. elegans*. *PLoS Genetics*.

[59] Zhang J., Bakheet R., Parhar R. S. (2011). Regulation of fat storage and reproduction by Kruppel-like transcription factor KLF3 and fat-associated genes in *Caenorhabditis elegans*. *Journal of Molecular Biology*.

[60] Dasgupta M., Shashikanth M., Gupta A. (2020). NHR-49 transcription factor regulates immunometabolic response and survival of *Caenorhabditis elegans* during Enterococcus faecalis infection. *Infection and Immunity*.

[61] Lemasters J. J., Qian T., He L. (2002). Role of mitochondrial inner membrane permeabilization in necrotic cell death, apoptosis, and autophagy. *Antioxidants & Redox Signaling*.

[62] Chacinska A., Koehler C. M., Milenkovic D., Lithgow T., Pfanner N. (2009). Importing mitochondrial proteins: machineries and mechanisms. *Cell*.

[63] Govindan J. A., Jayamani E., Ruvkun G. (2019). ROS-based lethality of *Caenorhabditis elegans* mitochondrial electron transport mutants grown on Escherichia coli siderophore iron release mutants. *Proceedings of the National Academy of Sciences of the United States of America*.

[64] Rolland S. G., Schneid S., Schwarz M. (2019). Compromised mitochondrial protein import acts as a signal for UPR^mt^. *Cell Reports*.

[65] Houtkooper R. H., Mouchiroud L., Ryu D. (2013). Mitonuclear protein imbalance as a conserved longevity mechanism. *Nature*.

[66] Ashrafi K. (2007). Obesity and the regulation of fat metabolism. *Worm Book*.

[67] Jones D., Dixon D. K., Graham R. W., Candido E. P. M. (1989). Differential regulation of closely related members of the hsp 16 gene family in *Caenorhabditis elegans*. *DNA*.

[68] Stringham E. G., Dixon D. K., Jones D., Candido E. P. (1992). Temporal and spatial expression patterns of the small heat shock (hsp 16) genes in transgenic *Caenorhabditis elegans*. *Molecular Biology of the Cell*.

[69] Wu Q., Zhao Y., Li Y., Wang D. (2014). Molecular signals regulating translocation and toxicity of graphene oxide in the nematode *Caenorhabditis elegans*. *Nanoscale*.

[70] Tower J. (2009). Hsps and aging. *Trends in Endocrinology and Metabolism*.

[71] Guerrero-Rubio M. A., Hernandez-Garcia S., Escribano J. (2020). Betalain health-promoting effects after ingestion in *Caenorhabditis elegans* are mediated by DAF-16/FOXO and SKN-1/Nrf2 transcription factors. *Food Chemistry*.

[72] Murphy C. T., Hu P. J. (2013). Insulin/insulin-like growth factor signaling in *C. elegans*. *Worm Book*.

[73] Christensen R., de la Torre-Ubieta L., Bonni A., Colón-Ramos D. A. (2011). A conserved PTEN/FOXO pathway regulates neuronal morphology during *C. elegans* development. *Development*.

[74] Accili D., Arden K. C. (2004). FoxOs at the crossroads of cellular metabolism, differentiation, and transformation. *Cell*.

[75] Wang C., An J., Bai Y. C. (2019). Tris(1,3-dichloro-2-propyl) phosphate accelerated the aging process induced by the 4-hydroxynon-2-enal response to reactive oxidative species in *Caenorhabditis elegans*. *Environmental Pollution*.

[76] Ye B., Rui Q., Wu Q., Wang D. (2010). Metallothioneins are required for formation of cross-adaptation response to neurobehavioral toxicity from lead and mercury exposure in nematodes. *PLoS One*.

[77] Holmstrom K. M., Finkel T. (2014). Cellular mechanisms and physiological consequences of redox-dependent signalling. *Nature Reviews. Molecular Cell Biology*.

[78] Mendelski M. N., Keshet A., Hoffschroeer N., Strieder T., Winter S. A., Paul R. J. (2019). ROS-mediated relationships between metabolism and DAF-16 subcellular localization in *Caenorhabditis elegans* revealed by a novel fluorometric method. *Cellular Signalling*.

[79] Mouchiroud L., Houtkooper R. H., Moullan N. (2013). The NAD^+^/sirtuin pathway modulates longevity through activation of mitochondrial UPR and FOXO signaling. *Cell*.

[80] Yang R. L., Rui Q., Kong L. (2016). Metallothioneins act downstream of insulin signaling to regulate toxicity of outdoor fine particulate matter (PM2.5) during Spring Festival in Beijing in nematode *Caenorhabditis elegans*. *Toxicology Research*.

