# Supplementary material for: Integrating Transcriptomics and Free Fatty Acid Profiling Analysis Reveal Cu Induces Shortened Lifespan and Increased Fat Accumulation and Oxidative Damage in C. elegans
Source: Oxid Med Cell Longev. 2022 Aug 16;2022:5297342. doi: 10.1155/2022/5297342 (PMC9398846; doi:10.1155/2022/5297342)
Supplement: Supplementary Materials — (1) Sample preparation, library construction, and RNA sequencing. (2) Bioinformatics analysis. (3) Detection of ROS and Mitochondrial membrane potential (MMP, Δψm). (4) Metabolite extraction and LC-MS/MS analysis. Figure S1: violin plot for all samples. Figure S2: correlation analysis for all samples. The correlation of each sample was above 0.97. Figure S3: GO enrichment analysis and top 10 pathways enrichment. Figure S4: mitochondrial ribosomal proteins were transcriptionally repressed by Cu. Table S1: the gene primer used in the study. Table S2: expression of differentially expressed genes (DEGs) in copper-exposed C. elegans. Table S3: top 12 statistics of pathways enrichment. Table S4: differential free fatty acid induced by 1 mg/L copper. Table S5: differential free fatty acid induced by 2 mg/L copper. [file 5297342.f1.doc]

Supplementary Materials for

**Integrating transcriptomics and free fatty acid profiling analysis reveals Cu shortened lifespan of *C. elegans* via fat accumulation and oxidative damage**

Ying Zhang, Qian Zhou, Lu Lu, Chao Zhao, Hu Zhang, Ran Liu, Yuepu Pu, Lihong Yin, *

*Corresponding author. Email: [lhyin@seu.edu.cn](mailto:xxxxx@xxxx.xxx)

This file includes:

Supplementary Text

Abstract Figure

Figs. S1 to S4

Tables S1 to S5

Supplementary Text

1. Sample preparation, library construction and RNA sequencing

When grown up to adult, C. elegans were collected to extract total RNA using Trizol reagent kit (Invitrogen, Carlsbad, CA, USA) according to the protocol. In brief, assess and check RNA quality using Agilent 2100 Bioanalyzer (Agilent Technologies, Palo Alto, CA, USA) and RNase free agarose gel electrophoresis. Then, enrich mRNA by Oligo(dT) beads, fragment mRNA into short fragments and reverse transcript into cDNA , synthesize the second strand cDNA. Purify the cDNA with QiaQuick PCR extraction kit(Qiagen, Venlo, The Netherlands), repair end, add poly(A), and ligate them to Illumina sequencing adapters. Finally, ligation products were size selected using agarose gel electrophoresis, PCR amplified, and sequenced using Illumina HiseqTM 6000 by Gene Denovo Biotechnology Co., Ltd (Guangzhou, China).

2. Bioinformatics analysis

Reads obtained from the sequencing machines, then filter of clean reads, align with ribosome RNA (rRNA) and reference genome, quantify gene abundance and DESeq2software was performed to analyze differential expression genes (DEGs) between two different groups. The offalse discovery rate (FDR) < 0.05 and absolute fold change ≥ 2 were considered differentially expressed genes/transcripts. Subsequently, Gene Ontology database (http://www.geneontology.org/) was conducted for GO terms analyses and Kyoto Encyclopedia of Genes and Genomes (KEGG) database was for identifying significantly enriched metabolic pathways or signal transduction pathways in DEGs. The calculated p-value was gone through FDR Correction, taking FDR ≤ 0.05 as a threshold.

3. Detection of ROS and Mitochondrial membrane potential (MMP, Δψm)

Fluorescent probe H2DCFDA (KeyGEN, Nanjing, China) was used to detected ROS level according to the manufacturer’s instructions. In brief, L4 stage N2 worms were treated with Cu until adult, washed worms three times, then 10 mM H2DCFDA was diluted to 10 μM using M9 solution, and worms were incubated by 10 μM H2DCFDA for 1 hour at 20 °C protected from light. The fluorescence was detected by the fluorescence microscope (Zeiss AX10, Germany).

JC-1 probe (Invitrogen, USA) was used to detected MMP. N2 worms were treated as above. Then, JC-1 dye was dissolved by freshly opened DMSO to 1 mg/mL stock solution, and diluted it to 2 μg/mL with M9 solution when using. Worms were stained with JC-1 for 1 hour at 20 °C protected from light. The MMP (Δψm) was detected by the fluorescence microscope (Zeiss AX10, Germany).

1. Metabolite extraction and LC-MS/MS analysis

The worm was extracted with 960 μL extracting solution (V_Isopropanol_ : V_n-Hexane_ = 2:3), add 40 μL internal standard (1 mg/L, in n-Hexane), vortex mixing for 10 s. Homogenized in ball mill for 4 min at 40 Hz, then ultrasound treated for 5 min (incubated in ice water). Centrifuge for 15 min at 12000 rpm, 4 °C. Transfer the supernatant 400 μL into a new 1.5 mL EP tubes, nitrogen blow dry. HPLC-grade methanol isopropanol, n-Hexane, and methanol were procured from CNW (Darmstadt, Germany). Add 200 μL Methanol and 100 μL (Trimethylsilyl) diazomethane (Macklin, Shanghai, China), vortex mixing for 10 s, stand at room temperature for 15 min, nitrogen blow dry, add 160 μL of n-Hexane and redissolve, centrifuge for 5 min at 12000 rpm, 4℃. Transfer the supernatant into a fresh vial for GC-MS analysis. An equivalent amount of each sample was prepared to serve as the quality control (QC) sample to monitor the stability and repeatability of the analytical process.

GC-MS analysis was performed using an Agilent 7890B gas chromatograph system (Agilent, California, USA) coupled with an Agilent 5977B mass spectrometer (Agilent, California, USA). The system utilized a DB-FastFAME capillary column. A 1 μL aliquot of the analyte was injected in split mode (5:1). Helium was used as the carrier gas, the front inlet purge flow was 3 mL min^−1^, and the gas flow rate through the column was 46 psi with constant pressure. The initial temperature was kept at 75 °C hold on 1 min; raised to 200 °C at a rate of 50°C min^−1^, hold on 1 min; raised to 210 °C at a rate of 2 °C min^−1^, hold on 1 min; raised to 230 °C at a rate of 10 °C min^−1^, hold on 16.5 min. The injection, transfer line, quad and ion source temperatures were 240 °C, 240 °C, 230 °C and 150 °C. The energy was -70 eV in electron impact mode. The mass spectrometry data were acquired in Scan/SIM mode with the m/z range of 33-400 after a solvent delay of 7 min.


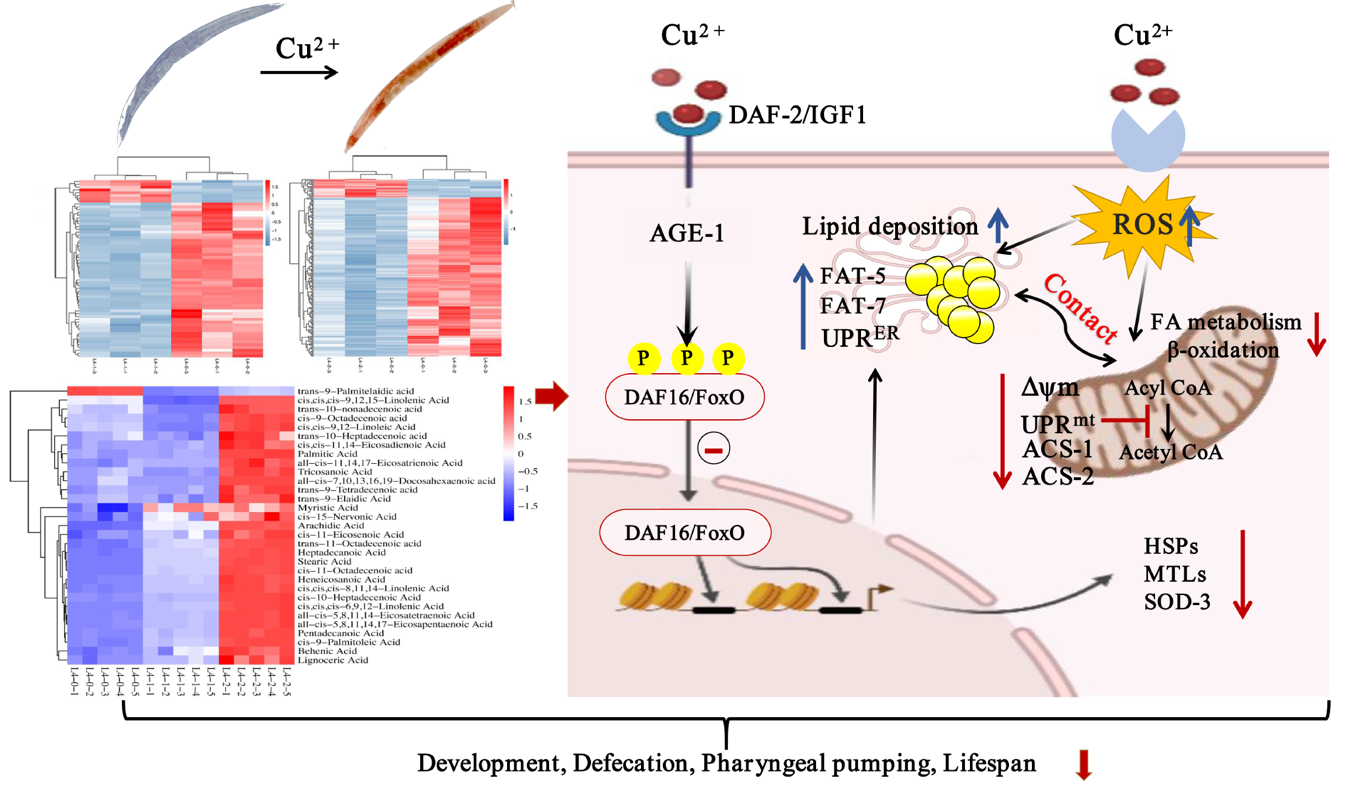


Abstract Figure

Fig. S1


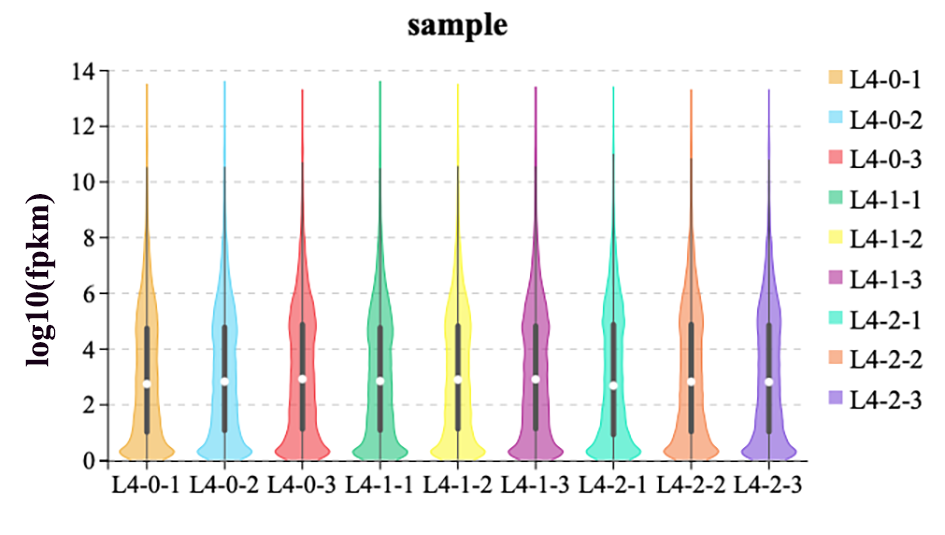


Fig. S1. Violin plot for all samples.

Fig. S2


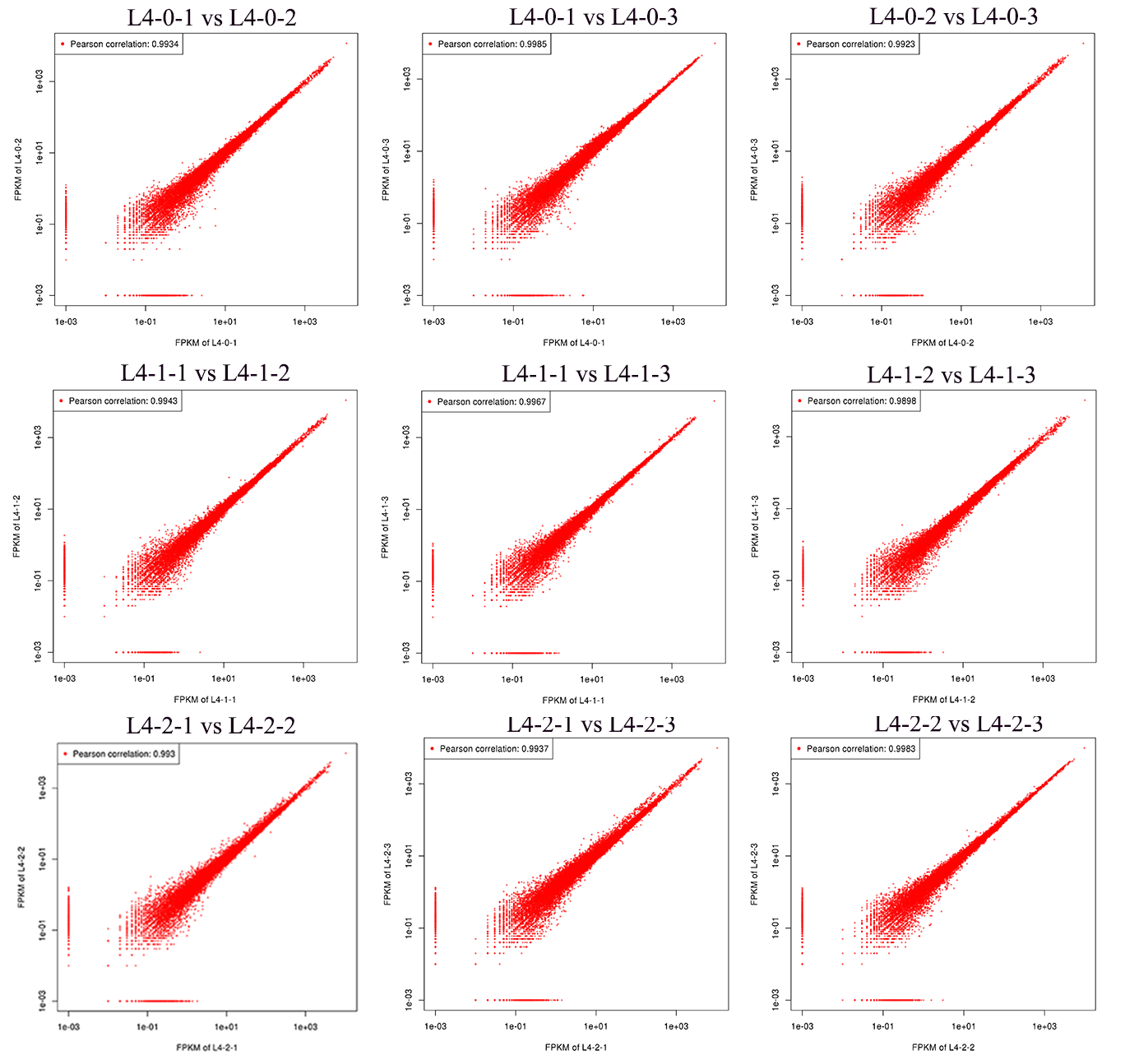


Fig. S2. Correlation analysis for all samples. The correlation of each sample was above 0.97.

Fig. S3


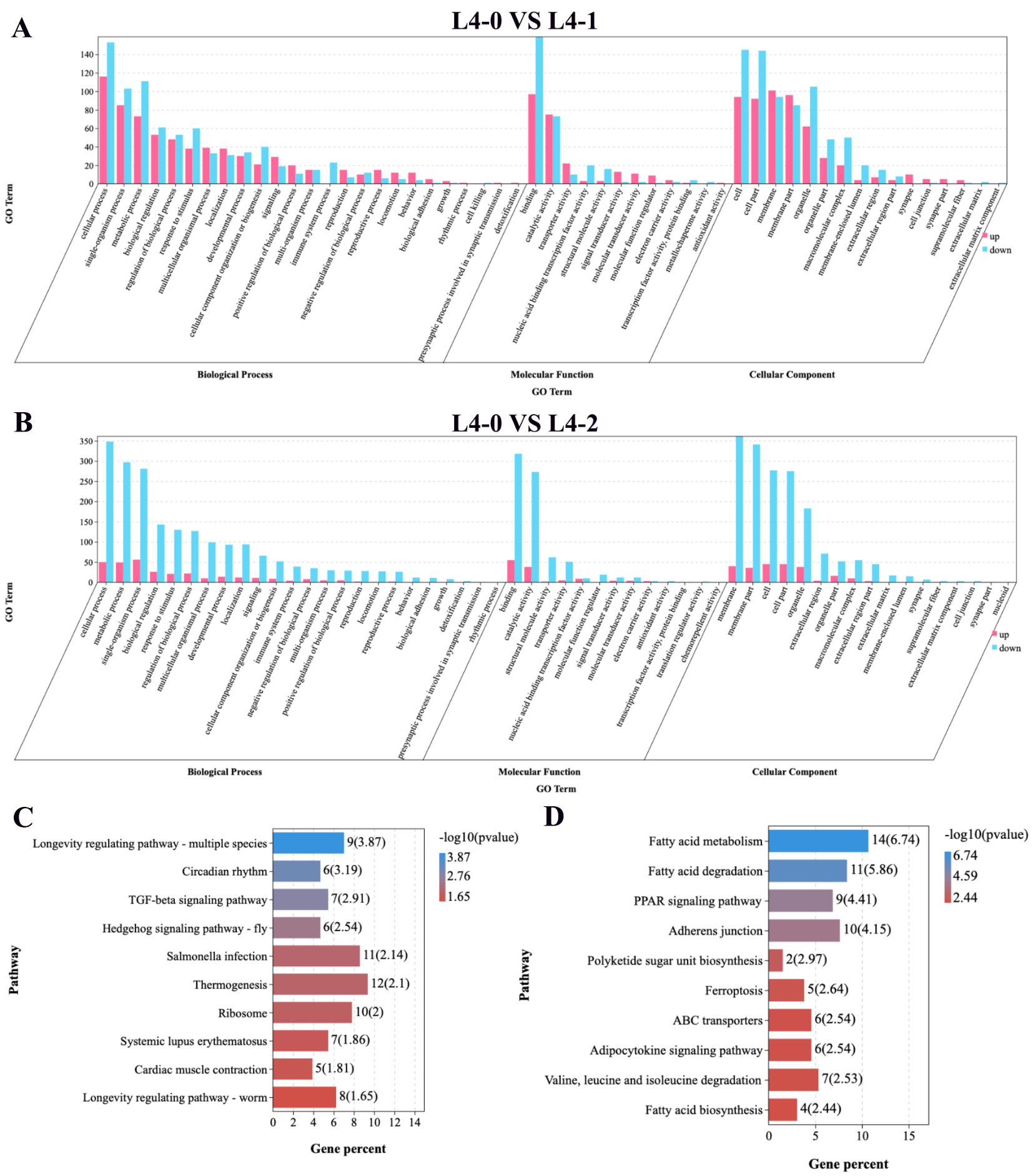


Fig. S3. GO enrichment analysis and top 10 pathways enrichment. (A)&(B): Major GO terms for C. elegans of Cu exposure. There were 25 GO terms in biological process (BP), 12 GO terms in molecular function (MF) and 13 GO terms in biological process (CC) of 0-VS-1 group. As for 0-VS-2 group, GO terms in BP, MF and CC of were 24, 16 and 17. (C )&(D): Top 10 pathways enrichment induced by Cu.

Fig. S4


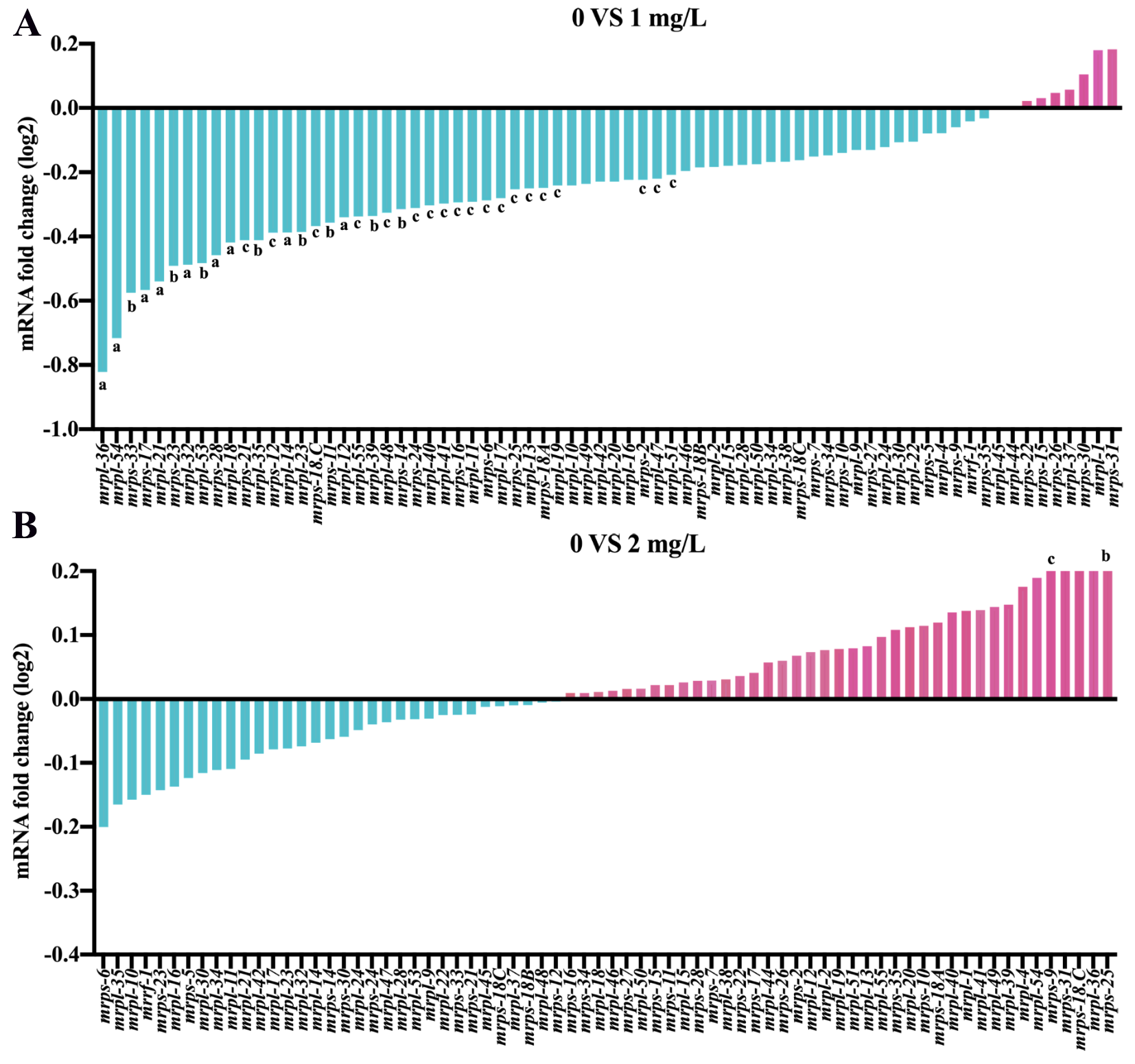


Fig. S4. Mitochondrial ribosomal proteins were transcriptionally repressed by Cu. (A) & (B) Expression of mitochondrial ribosomal protein in 0 VS 1 m/L and 0 VS 2 m/L respectively, a: P <0.001, b: P <0.01, c: P <0.05, compared with control.

**Table S1**

Table S1. The gene primer used in the study

| Gene Name | | Sequence (5’-3’) |
| --- | --- | --- |
| *acs-1* | Forward | TATCCACCACCACCAGTG |
|  | Reverse | ATACATAGGGTAGGGGGG |
| *acs-2* | Forward | GAGACTTTGGGACAGACA |
|  | Reverse | AAGTTCTTCGTGGAGGT |
| *age-1* | Forward | TCCTTGTTCACCTTGATGCTCGGA |
|  | Reverse | TTGACTGCGTGGAAGAGCCAATTC |
| *daf-2* | Forward | TCGAGCTCTTCCTACGGTGT |
|  | Reverse | CATCTTGTCCACCACGTGTC |
| *daf-16* | Forward | CCAGACGGAAGGCTTAAACT |
|  | Reverse | ATTCGCATGAAACGAGAATG |
| *fat-5* | Forward | TCATCAAGCCTCATCAGTAGAC |
|  | Reverse | CTCGGAAGTTCTATAGTCCTGG |
| *fat-7* | Forward | CGTAAGCATCCACAAGTTAAGG |
|  | Reverse | CAAAACAAAATGACCAGTGGGA |
| *β-actin* | Forward | CAAAATGTGTGACGACGAGG |
|  | Reverse | ATGACTCCTTGATGACGTGG |

**Table S2**

Table S2. Expression of differentially expressed genes (DEGs) in copper-exposed *C. elegans*

| Symbol | VS 1 mg/L  (FC)^#^ | VS 2 mg/L  (FC)^#^ | Description | Function |
| --- | --- | --- | --- | --- |
| acs-1 | 1.25^***^ | 0.53^***^ | Fatty Acid CoA Synthetase family | Involved in fatty acid metabolic process, enables medium-chain fatty acid-CoA ligase activity |
| acs-2 | 0.71^***^ | 0.41^***^ |
| acs-6 | 1.404^*^ | 0.510^***^ |
| acs-10 | 1.211^*^ | 0.630^***^ |
| fat-5 | 0.84 | 2.20^***^ | Delta(9)-fatty-acid desaturase fat-5 | Enables stearoyl-CoA 9-desaturase activity |
| fat-7 | 0.71^*^ | 5.20^***^ | Delta(9)-fatty-acid desaturase fat-7 |
| lipl-1 | 1.072 | 1.344^***^ | Lipase lipl-1 | Involved in fatty acid β-oxidation |
| abu-6 | 1.646^***^ | 0.561^***^ | Activated in Blocked Unfolded protein response | Involved in endoplasmic reticulum unfolded protein response |
| abu-7 | 1.484^***^ | 0.477^***^ |
| abu-8 | 1.445^***^ | 0.565^***^ |
| hsp-16.11 | 0.254^***^ | 1.671^***^ | Heat shock protein-16.1/Hsp-16.11 | IRE1-mediated unfolded protein response, determination of adult lifespan, endoplasmic reticulum unfolded protein response |
| hsp-16.41 | 0.181^***^ | 1.983^***^ | Heat shock protein-16.41 |
| hsp-16.48 | 0.272^***^ | 1.469^***^ | Heat shock protein-16.48 |
| hsp-16.49 | 0.272^***^ | 1.469^***^ | Heat shock protein-16.49 |
| hsp-70 | 0.325^***^ | 2.361^***^ | Heat Shock Protein | Enables heat shock protein binding, involved in adult lifespan |
| maco-1 | 1.328^**^ | 1.422^***^ | Macoilin | Involved in neuronal signal transduction |
| pept-1 | 1.219^**^ | 0.715^***^ | Peptide transporter family 1 | Iinvolved in lipid storage |
| pqn-57 | 1.608^***^ | 0.505^***^ | Prion-like-(Q/N-rich)-domain-bearing protein | Involved in endoplasmic reticulum unfolded protein response |
| pqn-74 | 1.358^***^ | 0.543^***^ | Chitin-binding type-2 domain-containing protein |
| rps-26 | 0.740^***^ | 0.731^***^ | 40S ribosomal protein S26 | Enables mRNA binding, involved in determination of adult lifespan |
| rps-28 | 0.645^***^ | 0.719^***^ | 40S ribosomal protein S28 | Enables structural constituent of ribosome |
| tcc-1 | 1.213^***^ | 1.271^***^ | Transmembrane and Coiled-Coil protein | Enables kinesin binding |
| asg-2 | 0.742^**^ | 0.753^**^ | Probable ATP synthase subunit g 2, mitochondrial | Part of mitochondrial proton-transporting ATP synthase complex, coupling factor F(o) |
| cox-7C | 0.666^***^ | 0.741^**^ | Cytochrome OXidase assembly protein | Involved in mitochondrial electron transport, cytochrome c to oxygen |
| ctc-1 | 1.543^***^ | 2.118^***^ | Cytochrome c oxidase subunit 1 | Mitochondrial electron transport, cytochrome-c oxidase activity, heme-copper terminal oxidase activity |
| ctc-3 | 1.577^***^ | 2.692^***^ | Cytochrome c oxidase subunit 3 |
| age-1 | 1.149^*^ | 1.189^*^ | Phosphatidylinositol 3-kinase age-1 | Enables 1-phosphatidylinositol-3-kinase activity, involved in insulin receptor signaling pathway and determination of adult lifespan |
| daf-2 | 1.283^***^ | 1.246 | Insulin-like receptor subunit beta; Receptor protein-tyrosine kinase | Involved in determination of adult lifespan and negative regulation of gene expression |
| daf-16 | 1.128 | 1.012 | Forkhead box protein O | Involved in cellular response to oxidative stress, determination of adult lifespan and insulin receptor signaling pathway, etc. |
| sod-1 | 0.807^**^ | 0.957 | Superoxide dismutase [Cu-Zn] | Enables copper ion binding and oxidoreductase activity,  involved in age-dependent response to oxidative stress and removal of superoxide radicals |
| sod-5 | 2.314 | 0.743 |
| sod-4 | 0.858 | 0.957 | Extracellular superoxide dismutase [Cu-Zn] |
| sod-2 | 0.802^***^ | 1.113 | Superoxide dismutase [Mn] 1, mitochondrial | Enables manganese ion binding and superoxide dismutase activity, removal of superoxide radicals, located in mitochondrion |
| sod-3 | 1.013 | 0.699 | Superoxide dismutase [Mn] 2, mitochondrial |
| mtl-1 | 0.450 | 0.986 | Metallothionein-1 | Involved in cellular response to metal ion |
| mtl-2 | 0.547 | 0.539 | Metallothionein-2 |

# FC: fold change. *: P<0.05, **: P<0.01, *** : P<0.001. Genes were sorted in order of appearance in the text.

**Table S3**

Table S3. Top 12 statistics of pathways enrichment

|  | KEGG A class | KEGG B class | Pathway | p |
| --- | --- | --- | --- | --- |
| NC VS 1 mg/L | Organismal Systems | Aging | Longevity regulating pathway - multiple species | 0.000 |
|  | Organismal Systems | Environmental adaptation | Circadian rhythm | 0.001 |
|  | Environmental Information Processing | Signal transduction | TGF-beta signaling pathway | 0.001 |
|  | Environmental Information Processing | Signal transduction | Hedgehog signaling pathway - fly | 0.003 |
|  | Human Diseases | Infectious diseases | Salmonella infection | 0.007 |
|  | Organismal Systems | Environmental adaptation | Thermogenesis | 0.008 |
|  | Genetic Information Processing | Translation | Ribosome | 0.010 |
|  | Human Diseases | Immune diseases | Systemic lupus erythematosus | 0.014 |
|  | Organismal Systems | Circulatory system | Cardiac muscle contraction | 0.015 |
|  | Organismal Systems | Aging | Longevity regulating pathway - worm | 0.022 |
|  | Cellular Processes | Cellular community - eukaryotes | Adherens junction | 0.023 |
|  | Metabolism | Xenobiotics biodegradation and metabolism | Drug metabolism - cytochrome P450 | 0.025 |
| NC VS 2 mg/L | Metabolism | Global and overview maps | Fatty acid metabolism | 0.000 |
|  | Metabolism | Lipid metabolism | Fatty acid degradation | 0.000 |
|  | Organismal Systems | Endocrine system | PPAR signaling pathway | 0.000 |
|  | Cellular Processes | Cellular community | Adherens junction | 0.000 |
|  | Metabolism | Metabolism of terpenoids and polyketides | Polyketide sugar unit biosynthesis | 0.001 |
|  | Cellular Processes | Cell growth and death | Ferroptosis | 0.002 |
|  | Environmental Information Processing | Membrane transport | ABC transporters | 0.003 |
|  | Organismal Systems | Endocrine system | Adipocytokine signaling pathway | 0.003 |
|  | Metabolism | Amino acid metabolism | Valine, leucine and isoleucine degradation | 0.003 |
|  | Metabolism | Lipid metabolism | Fatty acid biosynthesis | 0.004 |
|  | Human Diseases | Infectious diseases | Legionellosis | 0.004 |
|  | Organismal Systems | Aging | Longevity regulating pathway - multiple species | 0.016 |

**Table S4**

Table S4. Differential free fatty acid induced by 1 mg/L copper

| Name | FC | VIP* | P |
| --- | --- | --- | --- |
| Myristic acid (C14:0) | 1.13 | 1.06 | 2.11E-04 |
| Pentadecanoic acid (C15:0) | 1.11 | 1.09 | 3.98E-05 |
| Trans-9-Palmitelaidic acid (C16:1n7t) | 0.50 | 1.16 | 4.31E-13 |
| Cis-9-Palmitoleic acid (C16:1n7) | 1.10 | 1.13 | 1.12E-06 |
| Heptadecanoic acid (C17:0) | 1.17 | 1.16 | 5.62E-11 |
| Cis-10-Heptadecenoic acid (C17:1n7) | 1.09 | 1.15 | 1.09E-08 |
| Stearic acid (C18:0) | 1.11 | 1.16 | 8.76E-08 |
| Trans-11-Octadecenoic acid (C18:1n7t) | 1.29 | 1.15 | 5.29E-08 |
| Cis-9-Octadecenoic acid (18:1n9) | 0.95 | 1.14 | 2.25E-07 |
| Cis-11-Octadecenoic acid (C18:1n7) | 1.13 | 1.16 | 1.77E-11 |
| Cis, cis-9,12-Linoleic acid (C18:2n6c) | 0.93 | 1.15 | 1.15E-08 |
| Cis, cis, cis-6,9,12-Linolenic acid (C18:3n6) | 1.07 | 1.15 | 6.24E-09 |
| Cis, cis, cis-8,11,14-Linolenic acid (C18:3n4) | 1.09 | 1.15 | 2.93E-09 |
| Cis, cis, cis-9,12,15-Linolenic acid (C18:3n3) | 0.89 | 1.16 | 1.13E-10 |
| Trans-10-Nonadecenoic acid (C19:1n9t) | 0.87 | 1.09 | 7.62E-05 |
| Arachidic Acid (C20:0) | 1.20 | 1.16 | 1.49E-11 |
| Cis-11-Eicosenoic acid (C20:1) | 1.09 | 0.95 | 4.23E-03 |
| All-cis-5,8,11,14-Eicosatetraenoic Acid (C20:4n6) | 1.10 | 1.15 | 2.21E-09 |
| All-cis-5,8,11,14,17-Eicosapentaenoic Acid (C20:5n3, EPA) | 1.08 | 1.15 | 7.76E-08 |
| Heneicosanoic acid (C21:0) | 1.13 | 1.15 | 1.39E-08 |
| Behenic Acid (C22:0) | 1.13 | 0.89 | 1.16E-02 |
| Tricosanoic Acid (C23:0) | 0.97 | 0.73 | 4.38E-02 |
| Lignoceric Acid (C24:0) | 1.04 | 0.96 | 4.15E-03 |
| Cis-15-Nervonic Acid (C24:1) | 1.29 | 0.89 | 1.40E-02 |

# FC: fold change. * VIP values were obtained from OPLS-DA, whereas p values were obtained by comparison among multiple groups with the Kruskal-Wallis H test.

**Table S5**

Table S5. Differential free fatty acid induced by 2 mg/L copper

| Name | FC | VIP* | P |
| --- | --- | --- | --- |
| Myristic Acid (C14:0) | 1.13 | 0.95 | 5.03E-05 |
| Trans-9-Tetradecenoic acid (C14:1n5t) | 2.75 | 0.99 | 1.51E-07 |
| Pentadecanoic Acid (C15:0) | 1.59 | 1.01 | 2.05E-11 |
| Palmitic Acid (C16:0) | 1.27 | 1.01 | 2.30E-11 |
| Trans-9-Palmitelaidic acid (C16:1n7t) | 0.62 | 1.01 | 2.51E-10 |
| Cis-9-Palmitoleic Acid (C16:1n7) | 1.43 | 1.01 | 2.68E-12 |
| Heptadecanoic Acid (C17:0) | 1.61 | 1.01 | 5.00E-16 |
| Trans-10-Heptadecenoic acid (C17:1n7t) | 1.54 | 0.94 | 2.04E-04 |
| Cis-10-Heptadecenoic Acid (C17:1n7) | 1.60 | 1.01 | 6.56E-15 |
| Stearic Acid (C18:0) | 1.40 | 1.01 | 1.87E-14 |
| Trans-9-Elaidic Acid (C18:1n9t) | 1.47 | 1.00 | 6.83E-07 |
| Trans-11-Octadecenoic acid (C18:1n7t) | 1.99 | 1.01 | 1.03E-11 |
| Cis-9-Octadecenoic acid (18:1n9) | 1.21 | 1.01 | 8.94E-11 |
| Cis-11-Octadecenoic acid (18:1n7) | 1.50 | 1.01 | 9.63E-16 |
| Cis, cis-9,12-Linoleic Acid (C18:2n6) | 1.22 | 1.01 | 3.35E-10 |
| Cis, cis, cis-6,9,12-Linolenic Acid (C18:3n6) | 1.41 | 1.01 | 1.96E-13 |
| Cis, cis, cis-9,12,15-Linolenic Acid (C18:3n3) | 1.15 | 1.01 | 1.96E-07 |
| Cis, cis, cis-8,11,14-Linolenic Acid (C18:3n4) | 1.41 | 1.01 | 8.42E-12 |
| Trans-10-Nonadecenoic acid (C19:1n9t) | 1.38 | 0.99 | 5.95E-07 |
| Arachidic Acid (C20:0) | 1.50 | 1.01 | 2.23E-13 |
| Cis-11-Eicosenoic Acid (C20:1) | 1.36 | 1.00 | 2.74E-07 |
| Cis, cis-11,14-Eicosadienoic Acid (C20:2) | 1.46 | 1.00 | 6.83E-05 |
| Heneicosanoic Acid (C21:0) | 1.58 | 1.01 | 1.85E-07 |
| All-cis-5,8,11,14-Eicosatetraenoic Acid (C20:4n6) | 1.57 | 1.01 | 9.86E-13 |
| All-cis-11,14,17-Eicosatrienoic Acid (C20:3n3) | 1.38 | 1.00 | 9.20E-08 |
| Behenic Acid (C22:0) | 1.50 | 1.00 | 2.41E-08 |
| All-cis-7,10,13,16,19-Docosahexaenoic acid (C22:5n3, DPA) | 1.33 | 0.99 | 7.29E-05 |
| All-cis-5,8,11,14,17-Eicosapentaenoic Acid (C20:5n3, EPA) | 1.48 | 1.01 | 7.32E-13 |
| Tricosanoic Acid (C23:0) | 1.32 | 1.00 | 6.03E-08 |
| Lignoceric Acid (C24:0) | 1.22 | 0.98 | 4.73E-06 |
| Cis-15-Nervonic Acid (C24:1) | 1.56 | 0.95 | 1.83E-04 |

# FC: fold change. * VIP values were obtained from OPLS-DA, whereas p values were obtained by comparison among multiple groups with the Kruskal-Wallis H test.
